# LINC01137/miR-186-5p/WWOX: a novel axis identified from WWOX-related RNA interactome in bladder cancer

**DOI:** 10.3389/fgene.2023.1214968

**Published:** 2023-07-13

**Authors:** Damian Kołat, Żaneta Kałuzińska-Kołat, Katarzyna Kośla, Magdalena Orzechowska, Elżbieta Płuciennik, Andrzej K. Bednarek

**Affiliations:** ^1^ Department of Molecular Carcinogenesis, Medical University of Lodz, Lodz, Poland; ^2^ Department of Functional Genomics, Medical University of Lodz, Lodz, Poland

**Keywords:** WWOX, LINC01137, miR-186, long non-coding RNA, microRNA, cap analysis gene expression sequencing, bioinformatics, bladder cancer

## Abstract

**Introduction:** The discovery of non-coding RNA (ncRNA) dates back to the pre-genomics era, but the progress in this field is still dynamic and leverages current post-genomics solutions. WWOX is a global gene expression modulator that is scarcely investigated for its role in regulating cancer-related ncRNAs. In bladder cancer (BLCA), the link between WWOX and ncRNA remains unexplored. The description of AP-2α and AP-2γ transcription factors, known as WWOX-interacting proteins, is more commonplace regarding ncRNA but still merits investigation. Therefore, this *in vitro* and *in silico* study aimed to construct an ncRNA-containing network with WWOX/AP-2 and to investigate the most relevant observation in the context of BLCA cell lines and patients.

**Methods:** RT-112, HT-1376, and CAL-29 cell lines were subjected to two stable lentiviral transductions. High-throughput sequencing of cellular variants (deposited in the Gene Expression Omnibus database under the GSE193659 record) enabled the investigation of WWOX/AP-2-dependent differences using various bioinformatics tools (e.g., limma-voom, FactoMineR, multiple Support Vector Machine Recursive Feature Elimination (mSVM-RFE), miRDB, Arena-Idb, ncFANs, RNAhybrid, TargetScan, Protein Annotation Through Evolutionary Relationships (PANTHER), Gene Transcription Regulation Database (GTRD), or Evaluate Cutpoints) and repositories such as The Cancer Genome Atlas (TCGA) and Cancer Cell Line Encyclopedia. The most relevant observations from cap analysis gene expression sequencing (CAGE-seq) were confirmed using real-time PCR, whereas TCGA data were validated using the GSE31684 cohort.

**Results:** The first stage of the whole study justified focusing solely on WWOX rather than on WWOX combined with AP-2α/γ. The most relevant observation of the developed ncRNA-containing network was LINC01137, i.e., long non-coding RNAs (lncRNAs) that unraveled the core network containing UPF1, ZC3H12A, LINC01137, WWOX, and miR-186-5p, the last three being a novel lncRNA/miRNA/mRNA axis. Patients’ data confirmed the LINC01137/miR-186-5p/WWOX relationship and provided a set of dependent genes (i.e., *KRT18*, *HES1*, *VCP*, *FTH1*, *IFITM3*, *RAB34*, and *CLU*). Together with the core network, the gene set was subjected to survival analysis for both TCGA-BLCA and GSE31684 patients, which indicated that the increased expression of *WWOX* or *LINC01137* is favorable, similar to their combination with each other (*WWOX*↑ and *LINC01137*↑) or with *MIR186* (*WWOX*↑/*LINC01137*↑ but *MIR186*↓).

**Conclusion:** WWOX is implicated in the positive feedback loop with LINC01137 that sponges WWOX-targeting miR-186-5p. This novel WWOX-containing lncRNA/miRNA/mRNA axis should be further investigated to depict its relationships in a broader context, which could contribute to BLCA research and treatment.

## 1 Introduction

Even though the discovery of non-coding RNAs (ncRNAs) dates back to the pre-genomics era, the progress in this field is still dynamic. The current knowledge is three decades away from identifying the first microRNA (miRNA) or long non-coding RNA (lncRNA); the ncRNA-related field has thus achieved a relevant maturity and can leverage current post-genomics solutions. Nowadays, ncRNAs are classified as housekeeping or regulatory RNAs. Housekeepers include, e.g., ribosomal RNA (rRNA), small nuclear RNA (snRNA), and small nucleolar RNA (snoRNA), whereas regulatory ncRNAs encompass, e.g., miRNA, lncRNA, piwi-interacting RNA (piRNA), and circular RNA (circRNA) (Zhang P. et al., 2019). The implication of ncRNAs in human pathologies has been demonstrated in cardiovascular and neurological disorders or cancer (Lekka and Hall, 2018). For the latter, both the diagnosis and therapy benefit from using ncRNAs, such as miRNA, piRNA, and lncRNA (Le et al., 2021).

In recent years, our research focused on investigating the interplay between WWOX, a global modulator of gene expression, and AP-2α/AP-2γ transcription factors. Even though some experimental data confirm the role of these three proteins in regulating RNA interactomes, no literature review provided a synopsis of the current knowledge of WWOX. In contrast, most of the AP-2α/γ-related literature was summarized by us a few years ago (Kolat et al., 2019). Although a dozen experimental articles make up the reasonable collection, more research will presumably appear in the nearest future, especially since the involvement of ncRNAs in cancer is still a hot topic (Liu et al., 2021; Garbo et al., 2022; Han et al., 2022; Jiang et al., 2022). In one of our previous studies, some interesting observations were noted for WWOX and AP-2α/γ based on the cap analysis gene expression sequencing (CAGE-seq) of the bladder cancer (BLCA) cell lines. However, in that project, we mainly focused on signaling pathways regulated by protein-encoding genes (Kolat et al., 2022). Given that the implication of WWOX/AP-2 in ncRNA networks merits further investigation, this study aimed at constructing RNA interactomes containing WWOX and AP-2α/γ and then investigating the most relevant observation using data from bladder cancer cell lines and patients affected by this disease. The first stage of the whole study justified that the investigation of ncRNA interactomes should be focused solely on WWOX rather than on WWOX combined with AP-2α/γ. Later stages of this research indicated that WWOX is a significant factor in prolonging patient survival both individually and in combination with other genes. Interestingly, there was no research on WWOX and ncRNAs in bladder cancer (Table 1), placing our study at the beginning of such literature.

**TABLE 1:** Synopsis of ncRNA-related mechanisms that entail WWOX expression changes in cancer.

Non-coding RNA	Tumor type	Mechanism (and references)
miR-29a-3p and miR-29b-3p (miRNAs)	Lung cancer	Alters methylation of the *WWOX* promoter and induces its re-expression via targeting mRNAs of Dnmt3 transferases (Fabbri et al., 2007)
miR-134-5p (miRNA)	Head and neck cancer	Targets *WWOX* mRNAs, inducing metastasis and oncogenicity (Liu et al., 2014)
	Lung cancer	Promotes cell growth and apoptosis via targeting *WWOX* mRNAs (Chen et al., 2015)
miR-214-3p (miRNA)	Osteosarcoma	Involves in the negative feedback loop with *WWOX*; additionally, WWOX inhibits miR-10b, for which no negative loop was confirmed (Gao et al., 2016)
	Nasopharyngeal carcinoma	Inhibits apoptosis but increases proliferation by targeting *WWOX* mRNAs (Han et al., 2020)
miR-146-5p (miRNA)	Triple-negative breast cancer	*WWOX* restoration upregulates miR-146-5p that directly modulates fibronectin levels and leads to reduced invasion and metastasis; afterward, its impact on metastasis was linked to SMAD3 targeting (Khawaled et al., 2019; Khawaled et al., 2020)
miR-153-3p (miRNA)	Hepatocellular carcinoma	Promotes the Wnt/β-catenin pathway via targeting *WWOX* mRNAs (Hua et al., 2015)
miR-24-3p (miRNA)	Non-small-cell lung cancer	Targets *WWOX* mRNAs and inhibits apoptosis but increases viability, proliferation, and invasiveness (Wang et al., 2018)
miR-187-5p (miRNA)	Cervical cancer	Targets *WWOX* mRNAs, inducing the migratory potential of cells (Hung et al., 2020)
miR-670-5p (miRNA)	Lung cancer	Targets *WWOX* mRNAs, promoting proliferation, migration, and invasion (Li H. X. et al., 2021)
miR-625-3p (miRNA)	Colorectal cancer	Promotes migration, invasion, and chemotherapeutic resistance via inhibiting the CELF2/WWOX pathway (Zhang Y. et al., 2022)
circMTO1 (circRNA)	Glioblastoma	Upregulates *WWOX* expression via miR-92 sponging, eventually inhibiting cell proliferation (Zhang X. et al., 2019)
WWOX-AS1 (lncRNA)	Hepatocellular carcinoma	Upregulates *WWOX* expression via miR-20b-5p sponging, eventually decreasing proliferation and migration, but increasing apoptosis (Xu et al., 2020)
PARTICLE (lncRNA)	Breast cancer	Interacts with Dnmt1, affecting the global methylome and increasing methylation of the CpG island within the *WWOX* gene (O'Leary et al., 2017)
TSLD8 (lncRNA)	Hepatocellular carcinoma	Physically interacts with WWOX protein to stabilize it, eventually diminishing cell migration and viability (Xu et al., 2019)

## 2 Materials and methods

### 2.1 Cell culture, stable transductions, and variant comparisons

The culture of bladder cancer cell lines, RT-112, HT-1376, and CAL-29 (purchased from Deutsche Sammlung von Mikroorganismen und Zellkulturen [DSMZ], Brunswick, Germany), has been fully described in previous studies (Kolat et al., 2021a; Kolat et al., 2021b). Briefly, the RT-112 cell line was cultured in RPMI-1640 medium, HT-1376 in high-glucose DMEM, and CAL-29 in DMEM; all cell lines were supplemented with FBS, L-glutamine, and antibiotic—antimycotic and incubated under humidified conditions. Stable transduction was obtained using lentiviral overexpression systems for WWOX and AP-2α/γ (Kolat et al., 2021a; Kolat et al., 2021b). The antibiotic clone selection of the first transduction (WWOX) was conducted using puromycin, whereas G418 was used for selection during the second transduction (AP-2α or AP-2γ). For each cell line, six stable transductants were obtained: control/control (KK); control/AP-2α↑ (KA); control/AP-2γ↑ (KC); WWOX↑/control (WK); WWOX↑/AP-2α↑ (WA); and WWOX↑/AP-2γ↑ (WC). To confirm the efficiency of transductions, proteins were isolated using the RIPA lysis buffer with the addition of sodium orthovanadate, phosphatase inhibitor cocktail, and phenylmethylsulfonyl fluoride (Santa Cruz Biotechnology, Dallas, TX, United States). Subsequently, blotting for WWOX, AP-2α, and AP-2γ was performed in duplicate for each cellular variant, as described previously (Kolat et al., 2021a; Kolat et al., 2021b). In the current study, five comparison types (i.e., KA/KK, KC/KK, WK/KK, WA/KK, and WC/KK) were initially adopted from the previous research (Kolat et al., 2022), but the comparison scheme was updated once the workflow focused on WWOX, given the promising results for this protein compared to AP-2α and AP-2γ. From the second stage of this study, cellular variants with WWOX overexpression (represented by WK, WA, and WC) were collectively compared to those without WWOX overexpression (represented by KK, KA, and KC; denoted as the “native WWOX” group). The bladder cancer cell lines were treated as biological replicates of a specific cellular variant to limit the impact of tumor heterogeneity, similar to our previous study (Kolat et al., 2022).

### 2.2 Isolation of RNA, preparation of the CAGE library, sequencing, and mapping

The Extracol reagent (EURx, Poland) was used to isolate total RNA, and extraction was performed according to the manufacturer’s protocol. The RNA integrity number (RIN) was determined using the Agilent 2100 Bioanalyzer (Agilent Technologies, Santa Clara, CA, United States); the quality threshold was set at RIN >7.0. The total RNA was then reverse-transcribed using random primers (CAGE Library Preparation Kit; K.K. DNAFORM, Yokohama, Japan). The 5′-cap-located ribose diols in RNAs were oxidized and biotinylated. Following cap-trapping, streptavidin beads enabled RNA/cDNA hybrid selection. RNA digestion using RNase I/H was followed by linker ligation to 5′-and 3′-cDNA ends, enabling the construction of double-stranded cDNA libraries. Sequencing was performed using the NextSeq 500 sequencer (Illumina, San Diego, CA, United States) using 75 nt single-end reads. Deposited data from the nAnTi-CAGE experiment are held under the accession number GSE193659 in the Gene Expression Omnibus (GEO) database. The quality of the obtained data was evaluated via the FastQC tool (v0.11.9). The Burrows–Wheeler Alignment tool (v0.7.17) was used for mapping the tags to the hg38 human genome. Unmapped reads were processed by implementing hierarchical indexing for spliced alignment of transcripts (v2.0.5). Following modified Paraclu (from the RECLU pipeline) for tag clustering, BEDTools (v2.12) was used to extract regions with a 90% overlap between replicates. Clusters that did not meet the requirements of the irreproducible discovery rate of ≥0.1 and >200 bp were omitted.

### 2.3 Acquisition of the ncRNA list and differential expression analysis

All currently known ncRNAs were acquired from the Human Genome Organization Gene Nomenclature Committee (HGNC). This list was applied to our GEO record (GSE193659) to analyze only ncRNA data in the subsequent step. Differential expression analysis (DEA) was performed via the limma package (v3.42.2), with the voom () function to make CAGE-seq data useful for limma. The calcNormFactors () function was used to preprocess data, and low-expressed genes were filtered out (tags with ≥5 counts per million in ≥1 library were retained). After the voom transformation, the model was fitted in limma using weighted least squares via the lmFit () function. The log_2_ fold-change (log_2_FC) values were obtained for each comparison via the makeContrasts () function with default parameters. Empirical Bayesian smoothing of standard errors preceded the extraction of differentially expressed genes (log_2_FC >1 and *p* < 0.05) via the topTable () function.

### 2.4 Intersection analysis, heatmap visualization, and multiple factor analysis

The Venn diagram was prepared using the venn R package (v1.9) and modified visually using Inkscape (v1.1) to include item labels. Heatmaps were visualized using the Heatmapper that utilizes ggplot2, d3heatmap, and gplot R packages (Babicki et al., 2016). An average linkage served as the clustering method, whereas Spearman’s rank correlation was applied as the distance measurement method. A dendrogram was visualized via clustering to rows and columns. The genes that were intersected using the Venn diagram and presented on the heatmap served as active variables in the multiple factor analysis (MFA) together with features representing WWOX/AP-2 expression statuses (native or overexpressed) among RT-112, HT-1376, and CAL-29 cell lines. Spatial partitioning across Dim1 and Dim2 components was visualized using FactoMineR and factoextra R packages.

### 2.5 RNA interactome visualization, Gene Ontology, and identification of relevant features

Genes identified from the differential expression analysis were visualized alongside WWOX/AP-2 in the form of a network using Cytoscape (v3.7), which served as a tool for collecting data from a few ncRNA-related databases: miRDB (Wong and Wang, 2015), Arena-Idb (Bonnici et al., 2018), miRNet (Chang et al., 2020), LncRRIsearch (Fukunaga et al., 2019), snoDB (Bouchard-Bourelle et al., 2020), and RISE (Gong et al., 2018). In order to acquire the most relevant interactions, these servers were employed with various parameters that are summarized in the following sentences. miRDB was searched using miRNA names; only targets with the highest score were selected (e.g., if the highest available target score on a scale of 1–100 was 99, only targets with this score were selected and not the targets with a score of ≤98). Arena-Idb was queried by ncRNA names, and all interactions were acquired due to their low amount (typically four targets per ncRNA). miRNet lacked the targets for most input genes, so a similar approach was followed as in Arena-Idb; the GraphML files were downloaded via the “ncRNAs” query list. LncRRIsearch was queried with Ensembl gene ID and an energy threshold of −16 kcal/mol. Together with snoDB, RISE was used to acquire data on RNA–RNA interactions for snoRNAs; databases were searched using snoRNA names that directly provided results in a tabular form.

The part of a network that was directly connected to WWOX/AP-2 (henceforth denoted as the “interconnected network”) was subjected to functional annotations via the Database for Annotation, Visualization, and Integrated Discovery (DAVID) (Sherman et al., 2022) and Protein Annotation Through Evolutionary Relationships (PANTHER) (Thomas et al., 2022). DAVID was employed with Benjamini–Hochberg (BH) adjusted *p*-values to search among the “GOTERM_BP/MF/CC_DIRECT” ontology and pathways from Reactome/KEGG/WikiPathways, whereas the PANTHER overrepresentation test was annotated only using “PANTHER pathways” with Fisher’s exact test. DAVID was also used to acquire the entire annotation clusters, among which the two best clusters were selected owing to their higher enrichment scores than the remaining clusters. At this study stage, the emphasis was put on WWOX, considering the results acquired through the methodological workflow so far (see Sections 3.1 and 3.2 for more details). Genes from the interconnected network were subjected to Multiple Support Vector Machine Recursive Feature Elimination (mSVM-RFE) (Duan et al., 2005) that enabled the selection of best features (i.e., genes) distinguishing WWOX-overexpressing cellular variants from those with native WWOX expression. Feature ranking was executed via svmRFE () with k-fold cross-validation (CV) of k = 10 to include a multiplicity of mSVM-RFE and halve.above = 100. In order to allow SVM fitting, the R package e1071 was included in the R environment. Top features across all folds were obtained using writeFeatures () with a list of genes ordered by the ascending AvgRank value (the lower the number, the better the features). The expression of the most relevant genes in cellular variants with different WWOX levels was visualized using the vioplot (v0.4.0) R package.

### 2.6 Insights into the interconnected network

One of the most relevant ncRNAs from mSVM-RFE (i.e., LINC01137) was selected as the initial point for further WWOX-related research since it was the most credible result to unravel the direct lncRNA/miRNA/mRNA axis that contains WWOX (see Section 3.2. for details). Primarily, the genomic location of LINC01137 was visualized through the UCSC Genome Browser (UCSC stands for the University of California, Santa Cruz). Transcription factors (TFs) that are top interactors of the WWOX protein were acquired from the literature (Hussain et al., 2018) and further investigated for their binding sites near the genomic location of *LINC01137* via the Gene Transcription Regulation Database (GTRD v20.06) (Yevshin et al., 2019). Similar to the previous methodology section, the gene expression was visualized using the vioplot R package; the genes in close proximity to the genomic location of *LINC01137*, as well as the WWOX-interacting TFs, were included. The ENCORI database (Li et al., 2014) was employed to perform pairwise RNA–RNA co-expression analysis between selected ncRNAs. Subsequently, the common function (top similarly regulated pathways) of the involved messenger RNA (mRNA)-encoding genes (data for ncRNAs were unavailable) was predicted via correlation-based gene set enrichment analysis (corGSEA) under the Correlation AnalyzeR server (Miller and Bishop, 2021) assisted by a few literature datasets, FANTOM6 database, and Molecular Signatures Database (MSigDB).

### 2.7 Real-time quantitative PCR

Total RNA was extracted using the Extracol reagent (EURx, Poland) according to the manufacturer’s guidelines. Using ImProm-II™ reverse transcriptase (Promega, Madison, WI, United States), 10 mg of total RNA was reverse-transcribed into cDNA. After diluting each cDNA sample with DEPC water to a total volume of 140 μL, 2 μL was used for real-time quantitative PCR (GoTaq^®^ qPCR Master Mix, Promega). Each cellular variant was measured in triplicate using the LightCycler^®^ 480 (Roche Applied Science) with initial denaturation at 95°C for 2 min, followed by 45 cycles of 95°C for 30 s and annealing at 60°C for 30 s. Primer sequences for references and genes of interest are presented in Supplementary Table S1. The amplification of specific transcripts was confirmed by the melting curves at the end of each PCR. The relative expression level was determined using appropriate references: *H3F3A*, *RPLP0*, and *RPS17* were used for protein-encoding genes (*UPF1*, *RBM22*, and *ZC3H12A*), whereas *U6* served as an endogenous control for the expression of selected ncRNAs (*LINC01137*, *MIR6732*, and *MIR186*). The Universal Human Reference RNA (Stratagene, La Jolla, CA, United States) was applied as a calibrator. LinRegPCR (v2021.2) was implemented to determine the baseline and measurements of real-time qPCR efficiency. The relative expression level was calculated using the Pfaffl method (Pfaffl, 2001).

### 2.8 Data acquisition of cell lines or patients, sample selection, and reducing multidimensionality

Promising results were observed after implementing the workflow from previous methodological sections that directed the study to certify the observations in a broader context, i.e., using data from additional BLCA cell lines and bladder cancer patients. The DepMap portal (depmap.org/portal/; accessed on 10 January 2023) served as the source of the Cancer Cell Line Encyclopedia (CCLE) dataset that includes files from RNA sequencing of tumor *in vitro* models. Data on bladder cancer were acquired from two files from the dataset: “CCLE_RNAseq_rsem_genes_tpm_20180929.txt” and “CCLE_miRNA_20180525.gct.” Cell lines featuring the intended expression profile (i.e., with higher expression of *WWOX* and *LINC01137* but lower *MIR186* expression) were selected using the UpSetR package, which provides a scalable matrix-based alternative to Venn diagrams for performing intersection analysis and presenting the size or properties of sets (Conway et al., 2017). A similar procedure was executed in terms of bladder cancer patients, the cohort of which was acquired from The Cancer Genome Atlas (TCGA) via the Broad Institute Firehose (gdac.broadinstitute.org/; accessed on 14 January 2023) and The Atlas of ncRNA in Cancer (TANRIC) (Li et al., 2015). Together with our GEO dataset (GSE193659), the filtered data from the CCLE (henceforth denoted as “CCLE-BLCA”) and TCGA (henceforth denoted as “TCGA-BLCA”) were analyzed using MFA (similar to Section 2.4.) to reduce the multidimensional expression data of genes from the representative gene sets (see Section 3.4. for details).

### 2.9 Validation cohort, gene signatures, and survival analysis

Except for the TCGA-BLCA cohort, the study workflow so far utilized data from cancer cell lines, making the patients’ data unconfirmed at this stage. Therefore, the validation cohort was searched, taking into account the presence of at least 50 patients and at least two clinical endpoints, such as overall survival (OS), disease-specific survival (DSS), or progression-free interval (PFI). These requirements were chosen to facilitate and enrich one of the subsequent steps, i.e., the survival analysis that was performed using survival and survminer R packages. Eventually, GSE31684 was selected as a validation cohort with regard to the MFA from the previous section; clinical and expression data were acquired from GEO. Survival endpoints were evaluated on patients from GSE31684 and TCGA-BLCA using optimal cutoff values for the expression of a single gene [via our in-house Evaluate Cutpoints tool (Ogluszka et al., 2019)] or a few genes at once combined into the signature (via the which() function and further survival R package). Hazard ratios were visualized using the forestmodel R package with default parameters.

### 2.10 Insights into the core network

TargetScan (McGeary et al., 2019) was employed to identify possible binding between *WWOX* mRNA and miRNA of interest. Once the database was searched using the option “enter a human gene symbol,” the targeting of *WWOX* mRNA was evaluated using a table containing, e.g., context++ score (CS) for each miRNA–mRNA pair. Subsequently, the ncFANs tool (Zhang et al., 2021) was used to investigate the pairing between lncRNAs and miRNAs, as well as to receive additional data on the co-expression and formation of RNA–DNA triple helices (together with the number of triplex-forming oligonucleotides [TFOs]). The server was accessed via the ncFANs-NET module and queried using the lncRNA of interest, i.e., LINC01137. The ncFANs-NET module was built using large-scale sequencing data from public databases; thus, it should be more robust and valid. The co-expression network was selected with the “overall cancer” method and correlation coefficient ≥0.5, whereas the centric regulatory network was built with a miRanda score of ≥140 to reveal potential miRNA response elements (MREs) in LINC01137. Selected MREs were examined using the RNAhybrid tool (Rehmsmeier et al., 2004) to estimate the interaction’s minimum free energy (MFE).

### 2.11 Statistical analysis

The normality of the distribution was determined by the Shapiro–Wilk test; the statistical significance was evaluated with an unpaired *t*-test or Mann–Whitney test. The results with a *p*-value less than 0.05 were considered statistically significant.

## 3 Results and discussion

**FIGURE 1:**
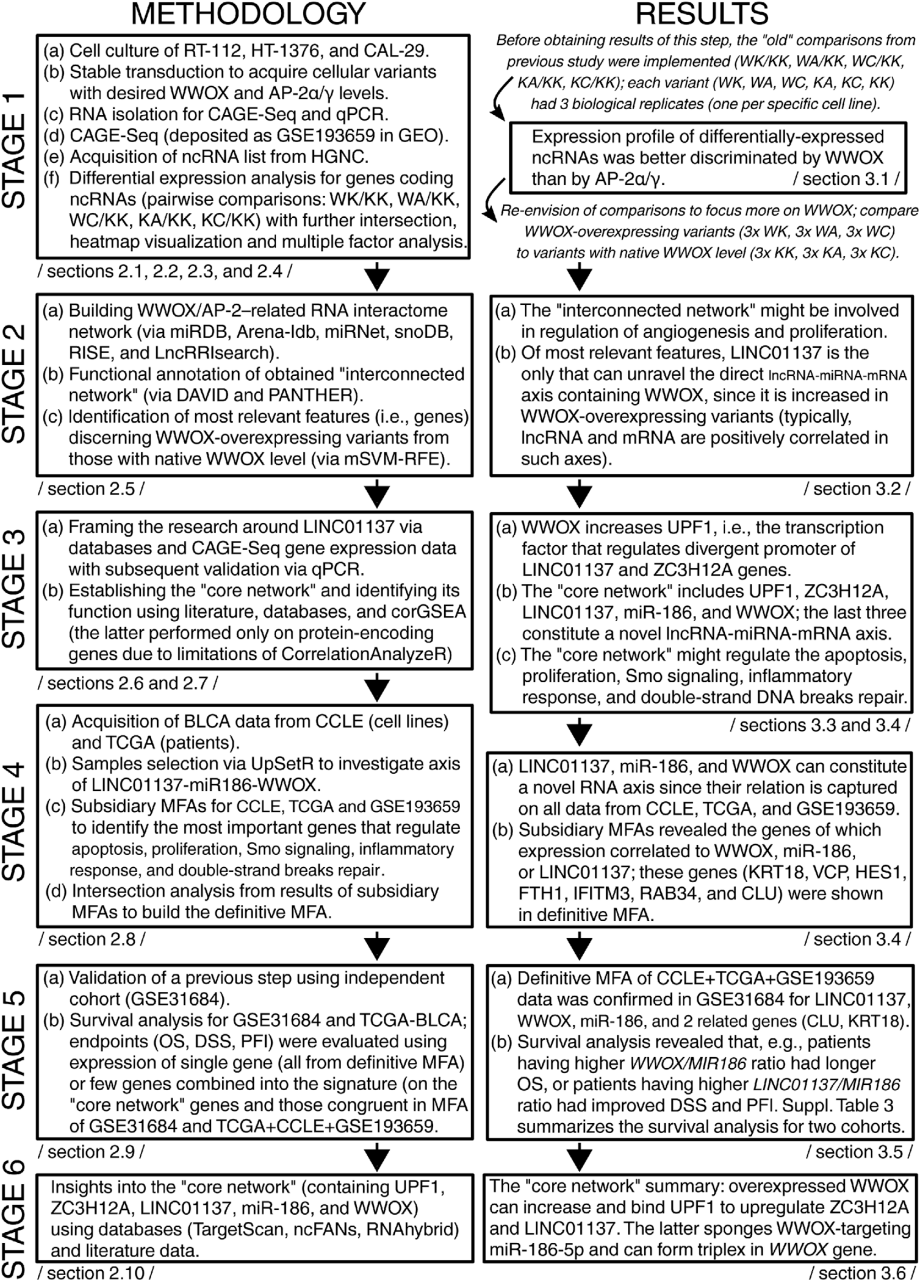
Overview of the methodology and results in consecutive stages of the current study. The first column represents the methodology, whereas the second summarizes the main results. Rows correspond to study stages. The workflow is depicted to facilitate the interpretation of a particular section. For each rectangle, the corresponding sections from the main text are indicated.

The methodology described previously is summarized alongside the main study findings in Figure 1. This allowed us to visualize which sections of methods or results correspond to each other and to which stage of the study they were assigned. The individual parts of the results and their discussion are presented in the following sections.

### 3.1 Groups were better discriminated by WWOX than by AP-2α/γ

**FIGURE 2:**
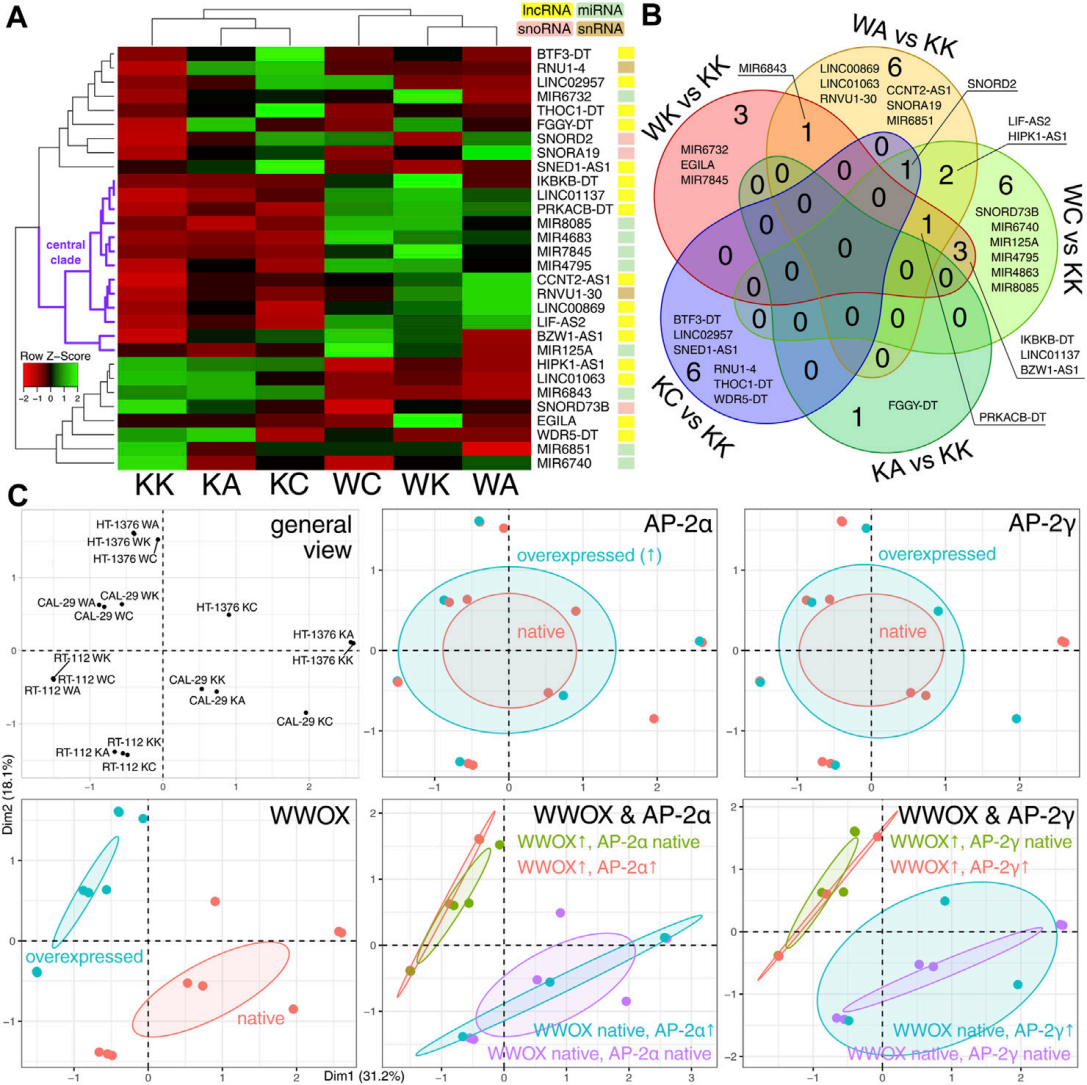
Initial comparison of cellular variants. **(A)** Differential expression analysis revealed that the expression profile of about half of the DEGs (central clade marked with purple) stably differed between WWOX-overexpressing variants (WK, WA, and WC) and those with native WWOX expression (KK, KA, and KC). **(B)** Intersection analysis suggested that WWOX might be superior to AP-2α/γ in regulating ncRNAs since the intersection between comparisons is present only for WK/KK, WA/KK, and WC/KK. **(C)** Multiple factor analysis ultimately revealed that the WWOX profile is more important for DEGs irrespective of whether analyzed alone or in combination with AP-2α/γ.

Initially, the differentially expressed ncRNA-encoding genes (DEGs) among cellular variants (KK, KA, KC, WK, WA, and WC) were visualized (Figure 2A), and their intersection for all pairwise comparisons [adapted from our previous research (Kolat et al., 2022)] was analyzed (Figure 2B). In total, 16 lncRNAs, nine miRNAs, three snoRNAs, and two snRNAs were identified. The central clade on the row dendrogram (marked with purple in Figure 2A) suggested that about half of DEGs stably depend on the presence of WWOX overexpression. The Venn diagram certified that WWOX might be superior to AP-2α/γ in regulating ncRNAs, the intersection between comparisons adapted from the previous research study, even if sparse, is only present for WK/KK, WA/KK, and WC/KK. The multidimensionality reduction provided a general view of the samples, which enabled grouping them based on the expression profile of WWOX and AP-2α/γ. Ultimately, this prompted us to focus solely on WWOX instead of WWOX combined with AP-2 [the latter was the case in the previous research (Kolat et al., 2022)] since the cellular variants were distinguished by WWOX regardless of whether it was analyzed alone or in combination with AP-2α/γ (Figure 2C). Even if the interaction between AP-2α/γ and ncRNAs is known (Kolat et al., 2019), this regulation might be subordinate to WWOX influence, as described specifically for AP-2α/γ (Kolat et al., 2021a) or regarding the general superiority of WWOX function (Pospiech et al., 2018). With regard to ncRNA data from our CAGE-seq experiment, it appears that investigating WWOX instead of AP-2α/γ is a relevant approach. Thus, the cellular variants with WWOX overexpression (represented by WK, WA, and WC) will be collectively compared to those without WWOX overexpression (represented by KK, KA, and KC) beginning from the consecutive part of the study (as shown in Figure 1).

### 3.2 LINC01137 was the most suitable for revealing the WWOX-containing lncRNA/miRNA/mRNA axis

**FIGURE 3:**
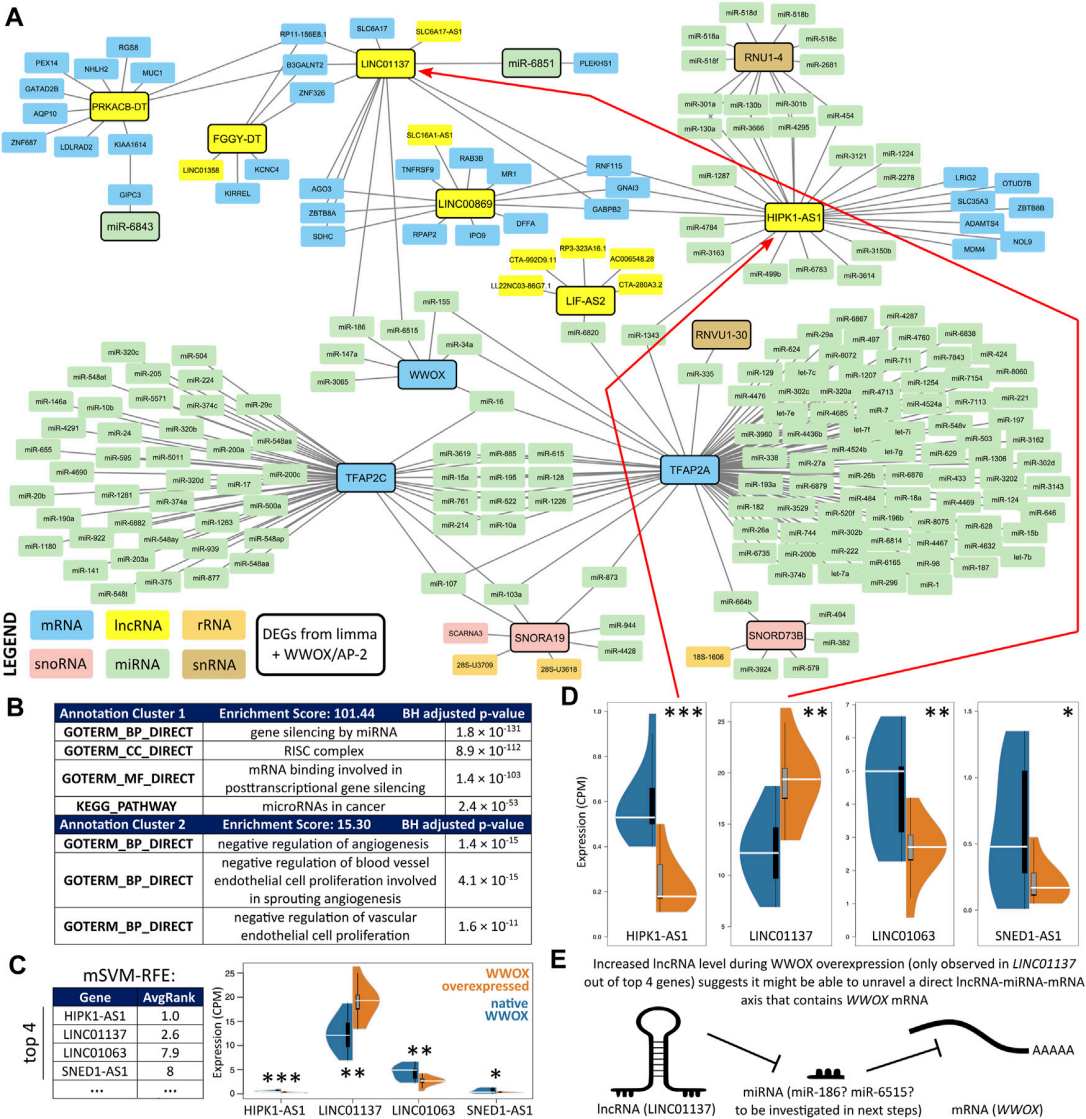
RNA interactome visualization. **(A)** An interconnection overview confirmed that 12 DEGs form a network with WWOX and AP-2α/γ (see Supplementary Figure S1 for the complete scheme with divided subnetworks). **(B)** Gene Ontology using annotation clusters revealed that the network might regulate angiogenesis and proliferation. **(C)** Recursive feature elimination indicated that the two best genes (*HIPK1-AS1* and *LINC01137*) are part of an interconnected network. **(D)** Expression differences of the top four DEGs from mSVM-RFE suggested that only *LINC01137* can directly reveal the WWOX-containing lncRNA/miRNA/mRNA axis since its expression increases in WWOX-overexpressing variants (i.e., WK, WA, and WC). **(E)** Synopsis that justifies proceeding with LINC01137-related investigation.

Elaboration of a network using the list of differentially expressed ncRNAs and various databases (see Section 2.5.) provided a comprehensive view of RNA interactomes (Figure 3A) for WWOX, as well as AP-2α and AP-2γ (these were also included to verify an assumption from the previous section, i.e., whether WWOX is more important than AP-2α/γ in discriminating DEGs). At first glance, some of the ncRNAs from the central clade on the row dendrogram (Figure 2A) generated a shared network with WWOX/AP-2 (only the part being an interconnected network is visible in Figure 3A; the complete scheme with divided subnetworks is shown in Supplementary Figure S1). The most prevalent group of ncRNAs regarded as the input (framed with a solid line) included lncRNAs, but some miRNAs, snoRNAs, and snRNAs were also present. Annotation clustering of Gene Ontology revealed that the gene expression regulation behind the interconnected network might be involved in angiogenesis and proliferation (Figure 3B); our cellular variants were previously confirmed to affect these two processes diversely (Kolat et al., 2021a; Kolat et al., 2022). The complete functional annotation is shown in Supplementary Table S2.

Subsequently, the entire network from Supplementary Figure S1 was examined to indicate the most relevant genes that distinguish WWOX-overexpressing variants (i.e., WK, WA, and WC) from those without WWOX overexpression (i.e., KK, KA, and KC). The mSVM-RFE algorithm ranked the genes according to their AvgRank value (the lower the number, the better the features), indicating that the top genes include *HIPK1-AS1*, *LINC01137*, *LINC01063*, and *SNED1-AS1*, which all encode lncRNAs (Figure 3C). Expression of these genes was shown on separate scales (Figure 3D), emphasizing that only the expression of *LINC01137* was elevated in WWOX-overexpressing variants. Moreover, *LINC01063* and *SNED1-AS1* were not a part of the interconnected network visualized in Figure 3A and had noticeably worse AvgRank values than *HIPK1-AS1* and *LINC01137*. Increased expression of *LINC01137* during WWOX overexpression suggests that LINC01137 might be able to unravel a direct WWOX-containing lncRNA/miRNA/mRNA axis (Figure 3E) because the expression of lncRNA and mRNA in such axes is oftentimes positively correlated (Pacholewska and Sung, 2019; Song et al., 2022). In summary, *LINC01137* was selected as the most relevant gene because a) it was a part of the interconnected network, b) it had been ranked as one of the best genes according to mSVM-RFE, c) its expression change between cellular variants was statistically significant, and d) it was increased in WWOX-overexpressing variants so that it might be able to unravel a direct WWOX-containing lncRNA/miRNA/mRNA axis. The following section is dedicated to identifying the mechanism by which WWOX upregulates *LINC01137*.

### 3.3 WWOX increases LINC01137 via UPF1, the protein that regulates the divergent promoter

**FIGURE 4:**
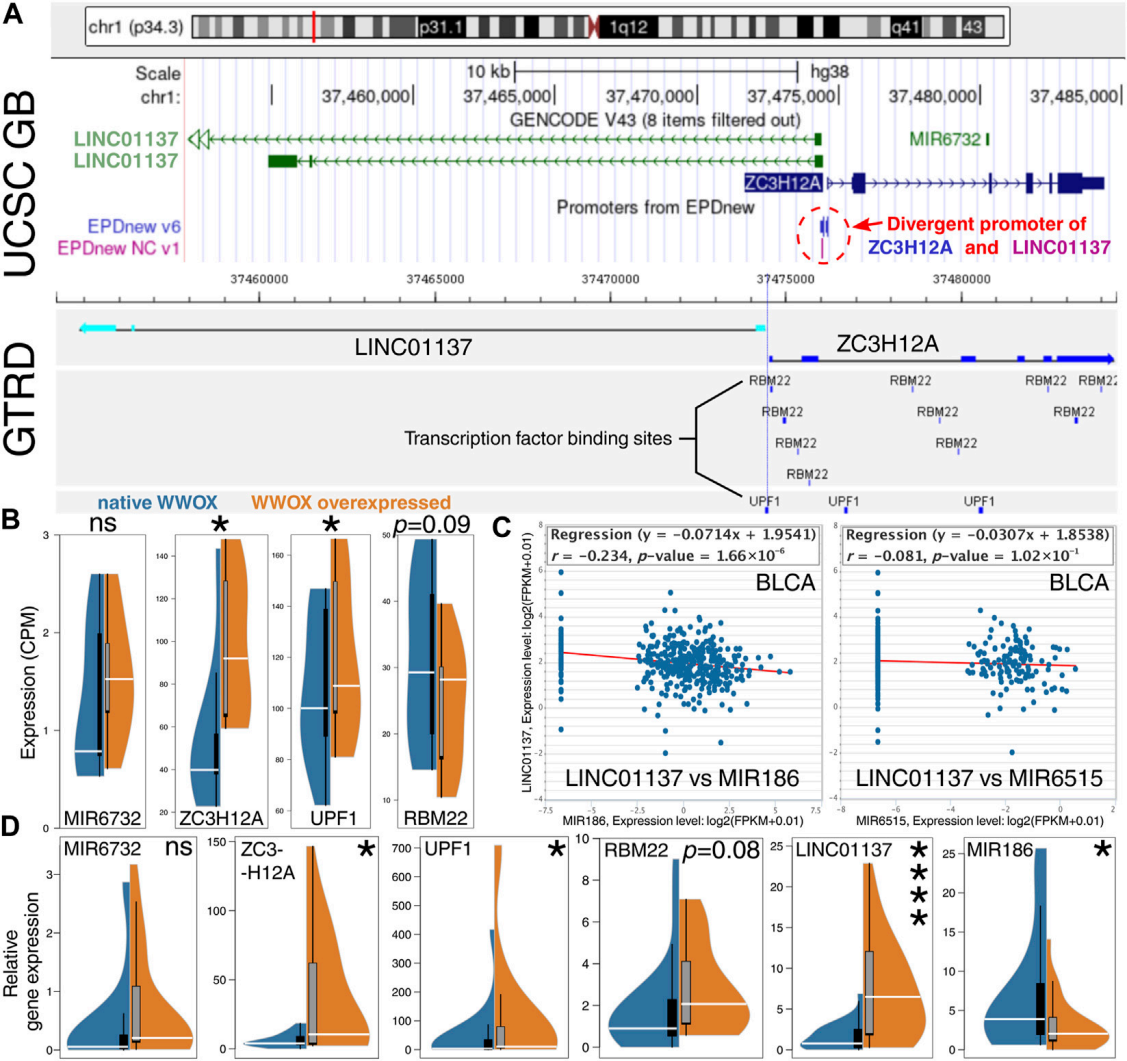
Insights into *LINC01137* and related genes or molecules. **(A)** The genomic location of *LINC01137* revealed that it shares a divergent promoter with *ZC3H12A* (top). Binding sites of two WWOX-interacting transcription factors, namely, RBM22 and UPF1, indicated that one binding site of UPF1 overlaps with a divergent promoter location (bottom). **(B)** Expression differences of investigated genes using CAGE-seq data suggested that UPF1 might be involved in WWOX-dependent upregulation of *LINC01137* and simultaneous RBM22-independent co-regulation of *ZC3H12A*. **(C)** Correlation between *LINC01137* and potentially related miRNAs (acquired from the interconnected network from Figure 3A) implies that *LINC01137* can be negatively correlated with *MIR186*. **(D)** Expression differences of investigated genes using real-time quantitative PCR data aligned with CAGE-seq findings.

Insights into the LINC01137–WWOX relationship began with an investigation of the genomic location of *LINC01137*. Based on the UCSC Genome Browser (Figure 4A, top panel), one can perceive that this locus is worth investigating since *LINC01137* is located near *MIR6732*, the miRNA-encoding gene that was also identified during the differential expression analysis (Figures 2A, B; WK vs*.* KK comparison). *LINC01137* shares the promoter with *ZC3H12A*, a gene encoded on the plus (or positive-sense) strand of DNA, and only *LINC01137* is encoded on the minus (or negative-sense) strand. Given that the sequence of both genes does not overlap, they are controlled under the so-called divergent promoter, i.e., a bidirectional region that enables the co-regulation of divergent transcription (Seila et al., 2009; Warman et al., 2021). It was justifiable to profoundly investigate the close proximity of the *LINC01137* locus with regard to the related genes (*MIR6732* and *ZC3H12A*) and transcription factors binding to this locus [as for TFs, our decision was to avail the protein interactome of WWOX (Hussain et al., 2018) to search among top interactors]. Two candidate transcription factors, RBM22 and UPF1, were evaluated for their binding sites near the divergent promoter (Figure 4A, bottom panel). Although binding sites of RBM22 were more prevalent, their localization overlapped with *ZC3H12A* and not *LINC01137*. At the same time, the RBM22 binding site that was the closest to *LINC01137* was outpaced by the binding site of UPF1 which completely overlapped with the location of a divergent promoter. This suggests that UPF1 might be involved in the WWOX-dependent upregulation of *LINC01137* and simultaneous RBM22-independent co-regulation of *ZC3H12A*. Similar to *LINC01137* (Figure 3D), our CAGE-seq data certified that *UPF1* and *ZC3H12A* are also significantly upregulated in WWOX-overexpressing cellular variants (i.e., WK, WA, and WC); however, no statistical significance was noted for *RBM22* and *MIR6732* (Figure 4B). Altogether, LINC01137 appears to be a good direction for further WWOX-related research studies, provided that it is possible to identify an miRNA that would complete the RNA axis. Candidate miRNAs were proposed based on the network visualized in Figure 3A; miR-186 and miR-6515 were found between LINC01137 and WWOX. After genes encoding these two miRNAs were correlated with *LINC01137* among BLCA patients, the statistically significant negative correlation between *LINC01137* and *MIR186* was noted (Figure 4C). In order to verify all observations described in this section, we decided to perform real-time PCR; the results were concordant for *LINC01137*, *MIR186*, *ZC3H12A*, and *UPF1* (Figure 4D). Although slight deviations were observed for *RBM22*, this did not affect the further study stages that focused entirely on ZC3H12A, UPF1, WWOX, LINC01137, and miR-186, especially the last three being an example of the lncRNA/miRNA/mRNA axis. Henceforth, the relationships between ZC3H12A, UPF1, WWOX, LINC01137, and miR-186 were denoted as the “core network.” Before focusing on the LINC01137/miR-186/WWOX axis, an additional enrichment analysis was performed for WWOX, UPF1, and ZC3H12A to predict their functional similarities (due to the limitations of Correlation AnalyzeR: only protein-encoding genes were investigated via this tool). Two annotations, namely, the double-strand break repair (DSBR) and smoothened (Smo) signaling pathway, were found to be repeated in the corGSEA of WWOX and UPF1, as well as WWOX and ZC3H12A (Supplementary Figure S2). Their usefulness in facilitating the workflow is explained in the following section.

### 3.4 Data from patients and additional cell lines confirm the relationship between WWOX, LINC01137, and miR-186, the latter being a missing element of the lncRNA/miRNA/mRNA axis

**FIGURE 5:**
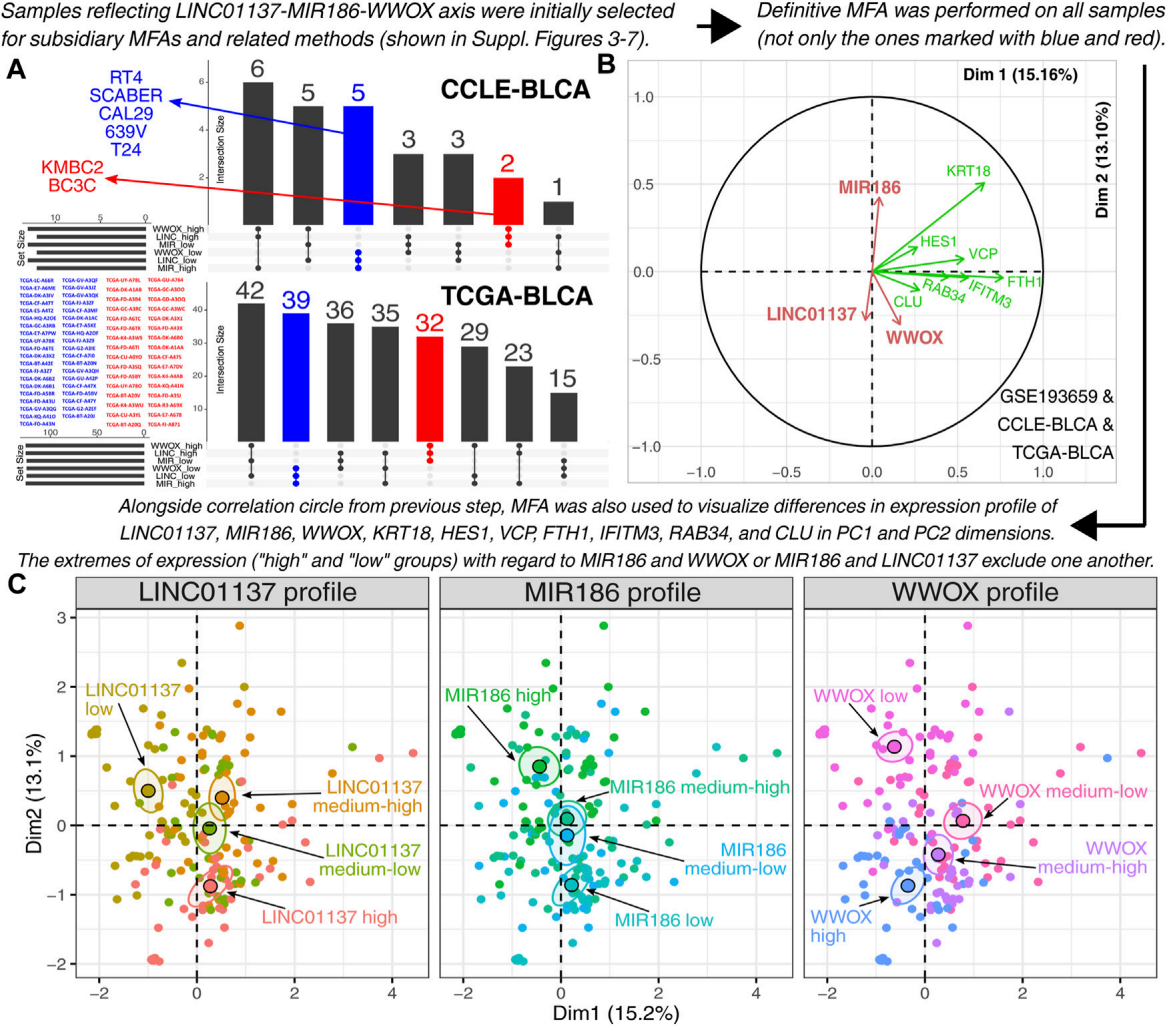
Investigation of the relationship between *LINC01137*, *MIR186*, and *WWOX*. **(A)** Relevant cell lines and patients from CCLE-BLCA and TCGA-BLCA were temporarily selected to facilitate the implementation of the definitive MFA (see Supplementary Figures S3–S7 for intermediate stages of the study workflow). **(B,C)** Definitive multiple factor analysis of patients and cell lines from CCLE-BLCA, TCGA-BLCA, and GSE193659 confirmed the positive correlation between *WWOX* and *LINC01137* and a negative correlation between *MIR186* and *WWOX* or *LINC01137*. All samples with various expression profiles, marked with black, blue, and red in subfigure **(A)**, are included in the last two subfigures.

The study was directed to certify the results in a broader context. In addition to RT-112, HT-1376, and CAL-29 cell lines, we decided to employ the CCLE and TCGA, which served as sources of BLCA data from cell lines and patients, respectively. To avoid excessive reduction of the group size, an emphasis was put on the LINC01137/miR-186/WWOX axis. The samples with specific expression profiles (i.e., high expression of *WWOX* and *LINC01137* but low *MIR186* expression; the second group with an opposite profile) were initially extracted. This enabled the selection of seven BLCA cell lines from the CCLE repository and 71 patients from TCGA-BLCA (Figure 5A). Afterward, each dataset was subjected to subsidiary MFA to identify the most relevant genes that explain the differences between opposite expression profiles (Supplementary Figure S3 for CCLE-BLCA; Supplementary Figure S4 for TCGA-BLCA; Supplementary Figure S5 for GSE193659). Five independent analyses were performed for each dataset; the number of analyses was dictated by separate biological processes that were included on the basis of the core network function. Thus, two corGSEA annotations related to ZC3H12A and UPF1 (i.e., DSBR and Smo signaling) were represented by the Reactome gene set R-HSA-5693606 and the MSigDB gene set M14890, respectively. The other three gene sets from MSigDB—M5902, M4627, and M10617—represented the apoptosis, proliferation, and inflammatory response, respectively, i.e., the processes that were determined on the basis of the literature data on core network genes. Namely, the interaction between ZC3H12A and UPF1 is known to orchestrate the cleavage of stem–loop-containing mRNAs during the early phase of inflammation (Mino et al., 2019). On the other hand, the role of miR-186 is widely described in terms of cell proliferation (Xiang et al., 2020). Data on LINC01137 functionality are scarce, which prompted us to access the FANTOM6 database and select the most significant annotation, i.e., the regulation of apoptosis; this is in line with a prominent role of WWOX in this process (Chen et al., 2021; Taouis et al., 2021). The intersection between datasets (Supplementary Figure S6) allowed us to identify genes that were further evaluated for their appropriateness in differentiating expression profiles (Supplementary Figure S7).

Ultimately, seven genes (i.e., *KRT18*, *HES1*, *VCP*, *FTH1*, *IFITM3*, *RAB34*, and *CLU*) met all requirements that stemmed from the analyses presented in Supplementary Figures S3–S7. These requirements were as follows: a) well explanation of the aforementioned expression profiles in CCLE-BLCA, TCGA-BLCA, and GSE193659 datasets; b) an intersection between all three datasets; and c) no distortion of the examined expression profile. Expression of the aforementioned genes (alongside *LINC01137*, *MIR186*, and *WWOX*) served as an input to the definitive MFA that was performed to summarize what GSE193659, CCLE-BLCA, and TCGA-BLCA share in common. Compared to subsidiary MFAs, all cell lines and patients were included in the definitive MFA (i.e., not only the one marked with blue or red in Figure 5A, but also the samples with heterogeneous expression profiles marked with black). Despite the inclusion of additional samples, the positive correlation between *WWOX* and *LINC01137* was still evident, alongside the negative correlation between *WWOX* and *MIR186* or *LINC01137* and *MIR186* (Figure 5B). The same is visible when distributing all samples within Dim1 and Dim2, with the best results for extremes of expression (Figure 5C).

### 3.5 Result validation is promising despite the scarcity of patients with a desired expression profile

**FIGURE 6:**
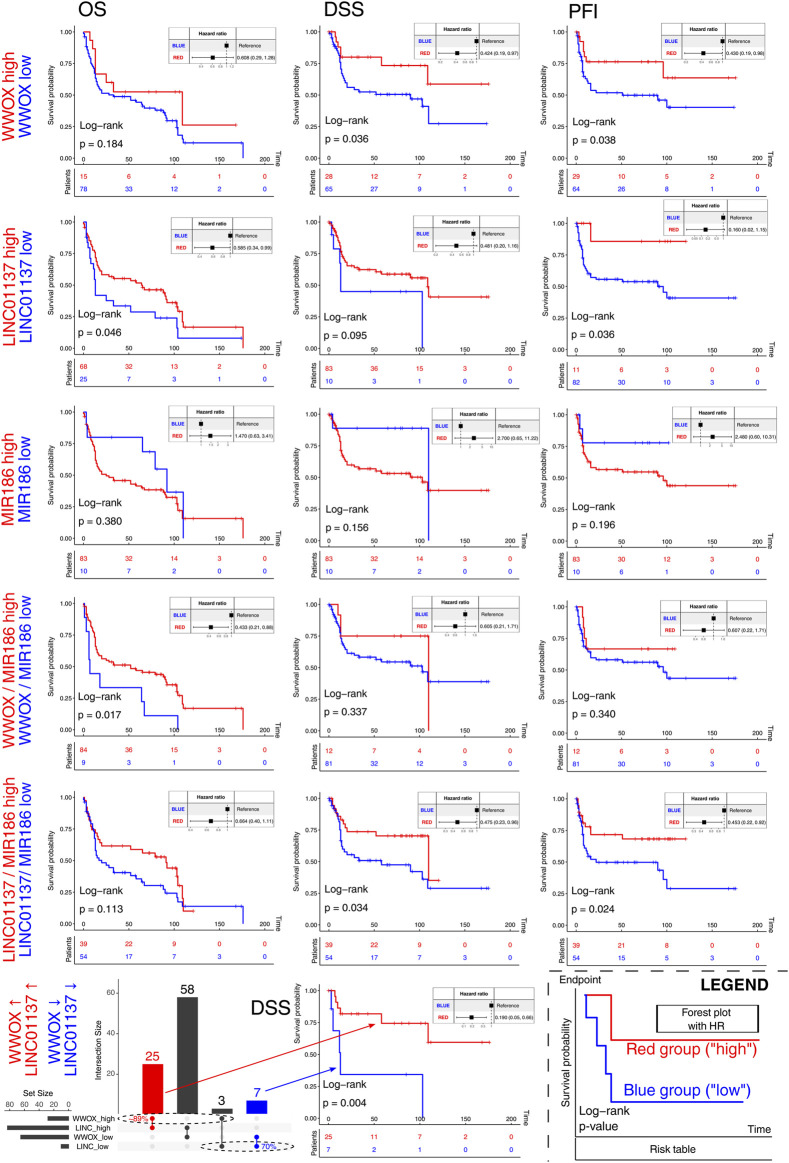
Survival analysis of *WWOX*, *LINC01137*, *MIR186*, and the related gene signatures. The bottom-right graph represents the figure’s legend. Investigated groups are marked with red and blue on the left side of each row. Except for the last row, the columns represent OS, DSS, and PFI. The last row visualizes an example of the UpSetR-created intersection of patients (DSS endpoint) having either high *WWOX* and high *LINC01137* (red) or the exact opposite (blue). A complete set of survival curves are visualized in Supplementary Figure S9 (TCGA-BLCA) and Supplementary Figure S10 (GSE31684), whereas the simplified summary with hazard ratios is presented in Supplementary Table S3.

Except for the TCGA-BLCA cohort, the study workflow so far has used data from cancer cell lines, making the verification of results on independent cohorts justifiable. The selection of the appropriate dataset (see Section 2.9. for details) revealed the GSE31684 cohort from the GEO, in which the definitive MFA from the previous section was validated. The results indicated that despite the lack of an evident correlation between *MIR186* and *LINC01137*, the *WWOX* gene still correlated positively with *LINC01137* and negatively with *MIR186* (Supplementary Figure S8). Together with *WWOX*, *LINC01137*, and *MIR186*, the remaining genes from the definitive MFA (i.e., *KRT18*, *HES1*, *VCP*, *FTH1*, *IFITM3*, *RAB34*, and *CLU*) were subsequently subjected to survival analysis that included not only the evaluation of each gene separately but also the gene signatures. As for the gene signatures, although we predominantly investigated various *WWOX*/*LINC01137*/*MIR186* expression combinations, a separate part was dedicated to the entire core network and two genes that presented congruent results in two MFA correlation circles (Figure 5B; Supplementary Figure S8): *KRT18* (positively correlated to *MIR186*) and *CLU* (positively correlated to *WWOX*). The entire set of Kaplan–Meier survival curves is visualized in Supplementary Figure S9 for TCGA-BLCA and Supplementary Figure S10 for GSE31684, whereas the simplified summary for both cohorts, including the hazards ratio, in a tabular form is shown in Supplementary Table S3. For instance, patients had superior OS when the expression of *WWOX*, *LINC01137*, *ZC3H12A*, and *UPF1* was high but *MIR186* expression was low, compared to the opposite profile (Supplementary Table S3; Supplementary Figure S9). For clarity and the given primary purpose of this section, only the data for *WWOX*, *LINC01137*, *MIR186*, and the related gene signatures are presented in Figure 6. High *WWOX* expression was found to prolong DSS and PFI; a similarity was found in terms of *LINC01137* and OS or PFI. It is also encouraging to note statistically significant results for the *WWOX*/*MIR186* ratio, where patients with a high ratio (i.e., with higher expression of *WWOX* but lower *MIR186*) survive longer than those with opposite ratios, as estimated using OS. Furthermore, a high *LINC01137/MIR186* ratio improved DSS and PFI. The gene/gene ratio was used for *MIR186* and either *WWOX* or *LINC01137* due to their supposedly opposite characteristics. However, in order to analyze the relationship between *WWOX* and *LINC01137*, we selected patients having either high or low expression of these two genes at once. The DSS-related example is visualized in the last row of Figure 6; the intersected patients constituted at least 70% of the reference group, a group that determined the maximum overlap due to its size. The remaining equivalents for OS and PFI are visualized in Supplementary Figures S9, S10.

The prognostic significance of WWOX has been evaluated in different tumors and various clinical endpoints (Zelazowski et al., 2011; Wen et al., 2017; Kaluzinska et al., 2021), including the statistically significant prediction of BLCA progression (Ramos et al., 2008), which aligns with our observations. Concerning LINC01137, the literature lacks data on BLCA (Li H. J. et al., 2021) and is inconsistent. For example, Huang and Huang (2021) proposed that LINC01137 was associated with poorer overall survival of patients with non-small-cell lung cancer. However, Lin et al. (2018) performed a prognostic meta-analysis on the same tumor, which revealed that LINC01137 is highly expressed in patients with low risk scores. Further research on this lncRNA is advisable, especially since the ratio of LINC01137 between tumor and normal specimens depends on the type of cancer tissue (Du et al., 2021). Lastly, the patient’s survival worsened when miR-186 was overexpressed in pancreatic ductal adenocarcinoma (Zhang et al., 2015) and esophageal cancer (Xue et al., 2021); however, no statistical significance was noted in luminal A breast cancer (Denkiewicz et al., 2019). As for BLCA, miR-186 was found to be the critical exosome-derived molecule that can induce natural killer cell dysfunction (Huyan et al., 2022), the process that entails the diminished production of effector cytokines and facilitates immune surveillance escape (Zhang W. et al., 2022).

### 3.6 Except for acting as an miR-186 sponge, LINC01137 can form an RNA–DNA triplex in WWOX

**FIGURE 7:**
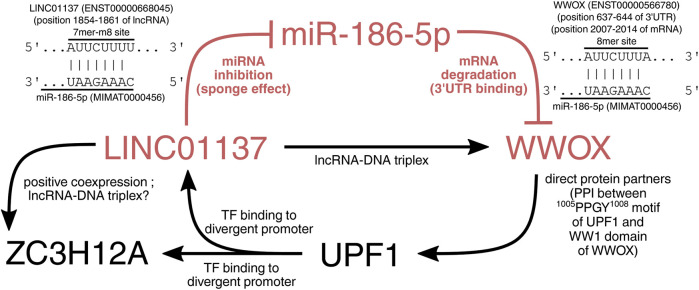
Visualization of the core network. The WWOX protein can interact with the UPF1 transcription factor to upregulate *ZC3H12A* and *LINC01137* genes. The latter gene encodes lncRNA that sponges miR-186-5p and recognizes *WWOX* mRNA. Moreover, LINC01137 is involved in a positive feedback loop with WWOX owing to its ability to form an RNA–DNA triplex in the genomic location of *WWOX*.

To ultimately demonstrate the relationship between *WWOX*, *LINC01137*, *MIR186*, and two other genes from the core network (i.e., *ZC3H12A* and *UPF1*), we employed a few tools and collected the available data from the literature. First, the direct interaction between WWOX and UPF1 proteins is possible via the proline-rich motif of UPF1 (^1005^PPGY^1008^) and the first tryptophan domain of WWOX (WW1) (Hussain et al., 2018). The recognition of proline-rich motifs by the WW1 domain is a common manner by which WWOX interacts with many protein partners (Abu-Odeh et al., 2014). Subsequently, in this network, UPF1 binds to the divergent promoter of *LINC01137* and *ZC3H12A*, as was investigated using GTRD. Expression changes in cellular variants suggest that UPF1 upregulates these two genes. Afterward, LINC01137 can act as an miRNA sponge, inhibiting the degradation of *WWOX* mRNAs carried out by miR-186. Nevertheless, there was still a need to specify which mature strand from the miR-186 stem–loop could target the mRNA of *WWOX* and whether the same strand can be sponged by LINC01137. The TargetScan database confirmed the possibility of miR-186-5p (but not -3p) binding to 3′-UTR of *WWOX* mRNA via ^5'^CAAAGAAU^3'^ and ^5'^AUUCUUUA^3'^, respectively (CS of −0.19). Since the seed site was stringent, one can infer that mRNA will be degraded (Saito and Saetrom, 2010; Mullany et al., 2016). On the other hand, the potential binding of miR-186 and LINC01137 with the specific sequence was not confirmed straightforwardly in any database. To compensate for this, we employed the ncFANs tool to calculate the miRanda score between LINC01137 and any miR-186 mature sequence, determining the possible region of their interaction. The results revealed that MREs for miR-186-5p (but not -3p) are present in the 1838–1861 positions of LINC01137 with a miRanda score of 150.00. Within this location (i.e., in the 1854–1861 position of lncRNAs), the RNAhybrid tool indicated the 7mer-m8 site of LINC01137 (^5'^AUUCUUUU^3'^) that matches the same sequence of miR-186-5p (^5'^CAAAGAAU^3'^) as WWOX. MFE of binding between LINC01137 and miR-186-5p was −16.6 kcal/mol [similar works accept a threshold of around −15 kcal/mol (Liu et al., 2013; Dandare et al., 2021)]. Since LINC01137 was the molecule that facilitated the unraveling of relationships, we employed other modules of the ncFANs tool to further investigate if any other dependencies with the rest of the core network exist. Interestingly, not only was *LINC01137* confirmed to co-express with *ZC3H12A* (r > 0.5; FDR <0.05; certifying their co-regulation under the divergent promoter), but it also forms the RNA–DNA triple helix (also known as triplex) in the genomic location of *WWOX* using 14 TFOs [typically, TFOs are 12–28 in length (Li et al., 2022)]. Such structures can repress or induce gene expression (Alecki and Vera, 2022; Cecconello et al., 2022; Warwick et al., 2023), but the latter can be assumed in our case. The module evaluating triple helices also returned one TFO within the *ZC3H12A* gene, which should be noted for future studies. In general, these aspects should be profoundly investigated by more sophisticated methods such as triplex-seq (Warwick et al., 2022). Altogether, relationships in the core network are visualized in Figure 7.

## 4 Conclusion

Determining the influence of WWOX on the ncRNA interactome in bladder cancer is still in its infancy; the subject merited investigation and was inaugurated by the current study. Following the construction of the ncRNA-containing network, various bioinformatics tools utilized the data from cell lines and patients, proposing a novel WWOX-related axis from the RNA interactome of bladder cancer. In this axis, WWOX is involved in the positive feedback loop with LINC01137, i.e., the lncRNA that sponges WWOX-targeting miR-186-5p. It is advisable to perform subsequent research to depict the relationships in a broader context, which may confer benefits to the clinic.

## Data Availability

The datasets presented in this study can be found in online repositories. The names of the repository/repositories and accession number(s) can be found at: https://www.ncbi.nlm.nih.gov/geo/; GSE193659.
