# Supplementary material for: LINC01137/miR-186-5p/WWOX: a novel axis identified from WWOX-related RNA interactome in bladder cancer
Source: Front Genet. 2023 Jul 13;14:1214968. doi: 10.3389/fgene.2023.1214968 (PMC10373930; doi:10.3389/fgene.2023.1214968)
Supplement: Supplementary file 1 [file Table1.DOCX]

Supplementary Material

LINC01137/miR-186-5p/WWOX: A Novel Axis Identified From WWOX-Related RNA Interactome in Bladder Cancer

Damian Kołat^1^*, Żaneta Kałuzińska-Kołat^1^, Katarzyna Kośla^1^, Magdalena Orzechowska^1^, Elżbieta Płuciennik^2^, Andrzej K. Bednarek^1^

*** Correspondence:** Damian Kołat: [damian.kolat@umed.lodz.pl](mailto:damian.kolat@umed.lodz.pl)

## Supplementary Figures

For the sake of high quality, all Supplementary Figures are also available as separate TIFF files.

**
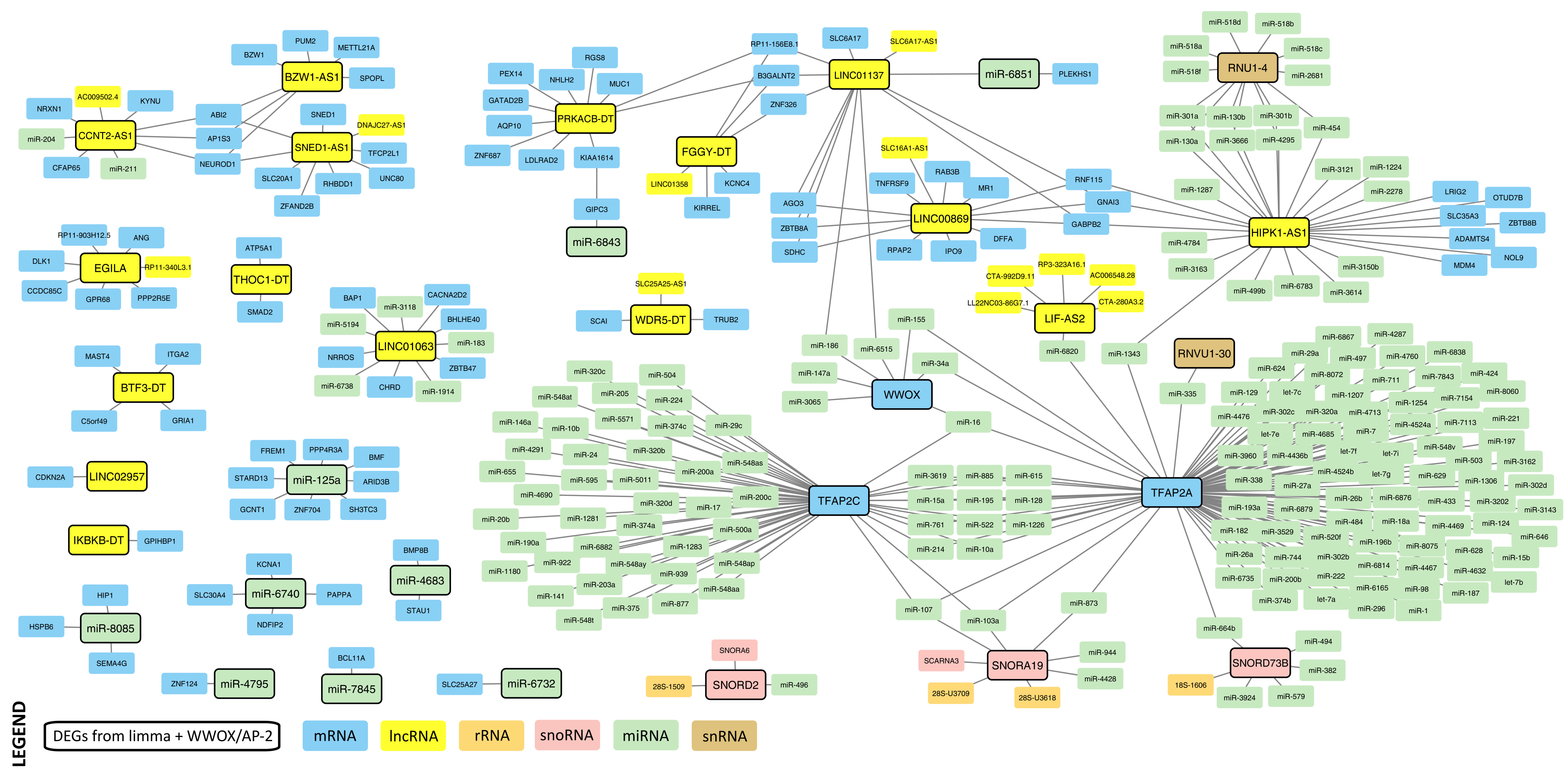
Supplementary Figure 1.** Interconnected network alongside separate subnetworks.


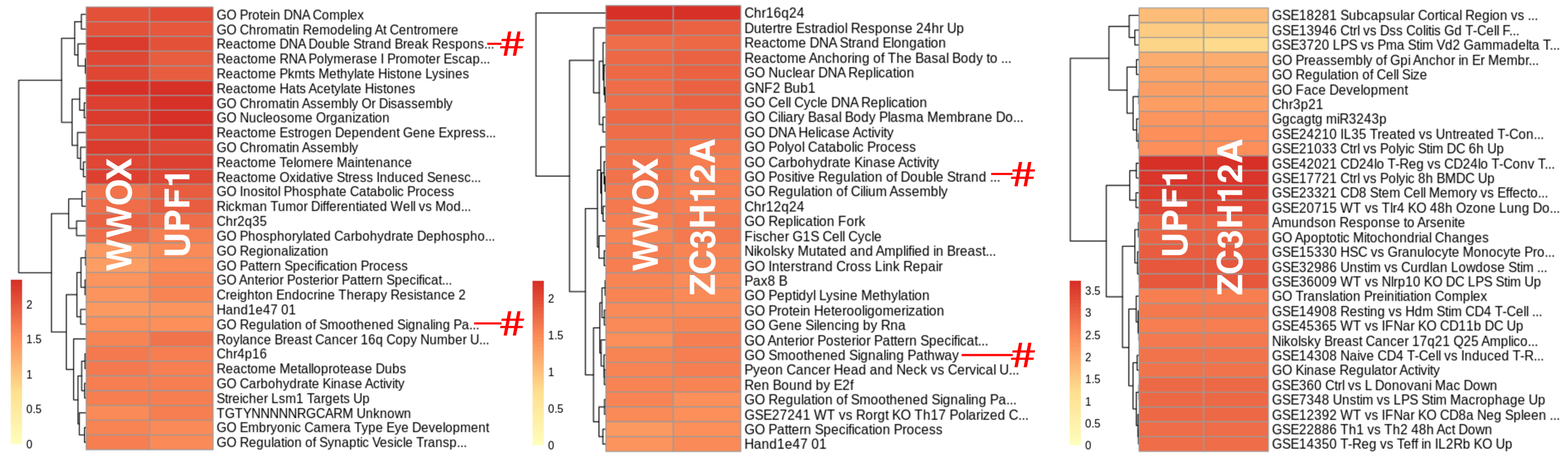


**Supplementary Figure 2.** Correlation-based gene set enrichment analysis of WWOX, UPF1, and ZC3H12A.


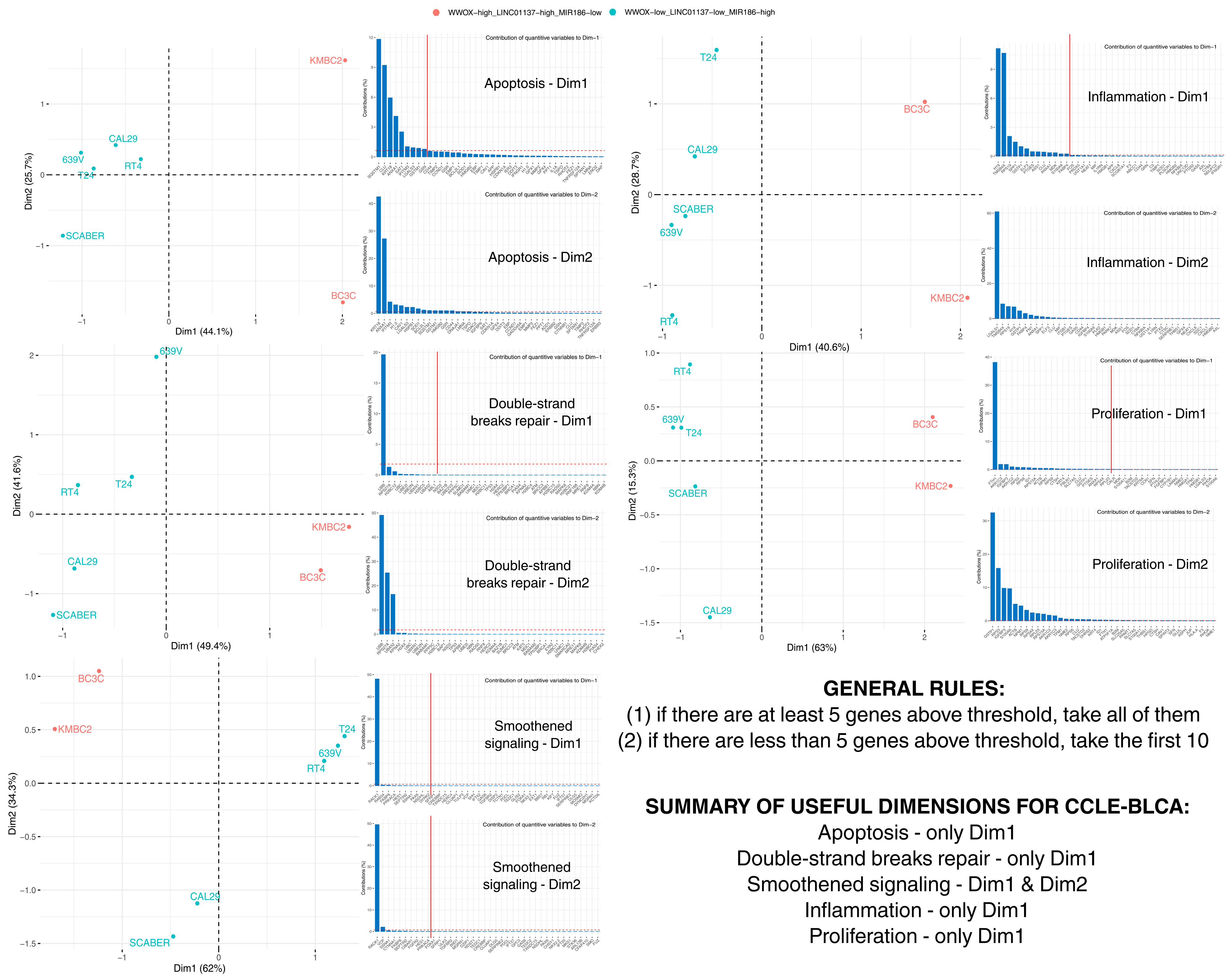


**Supplementary Figure 3.** Subsidiary MFA for CCLE-BLCA.


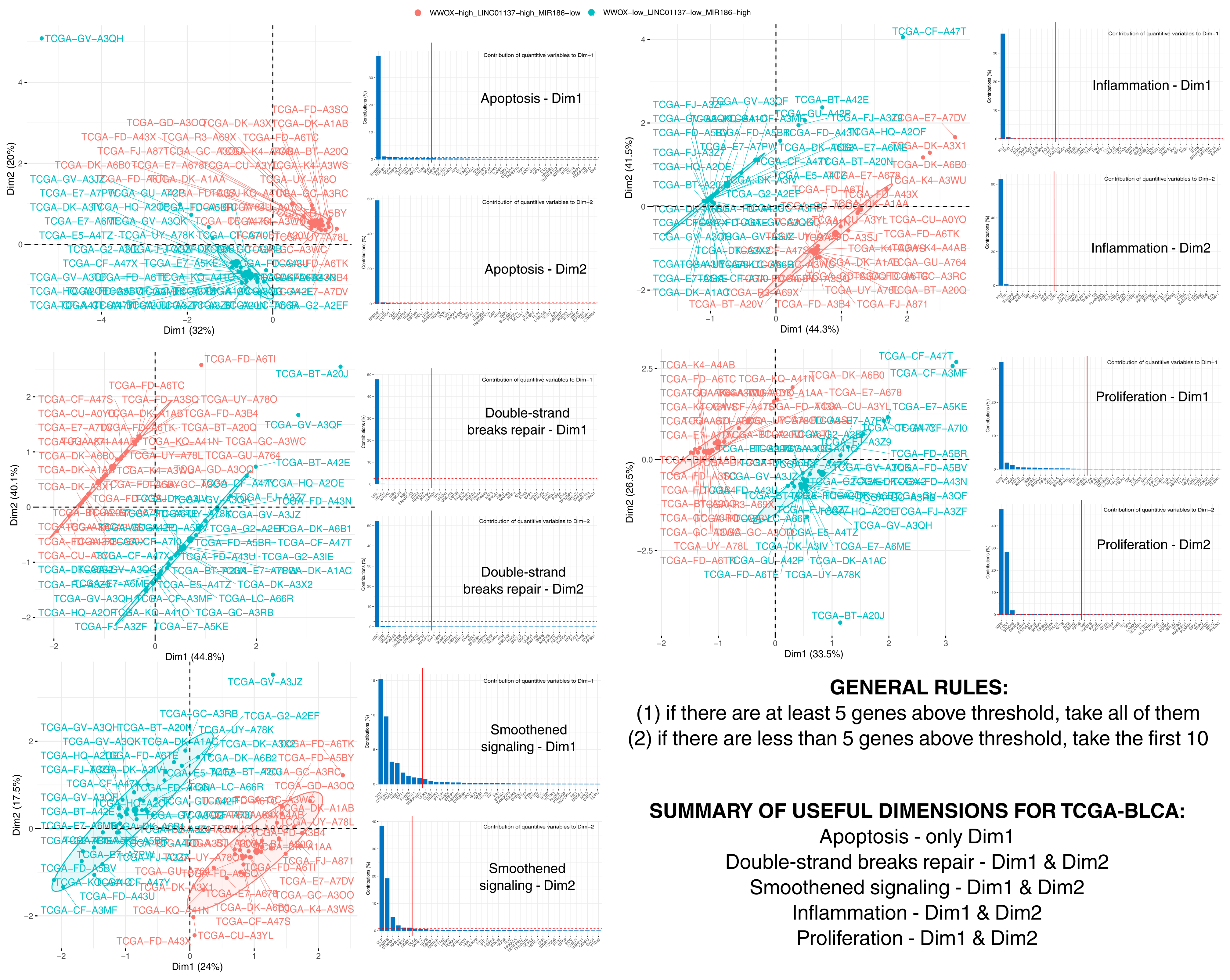


**Supplementary Figure 4.** Subsidiary MFA for TCGA-BLCA.


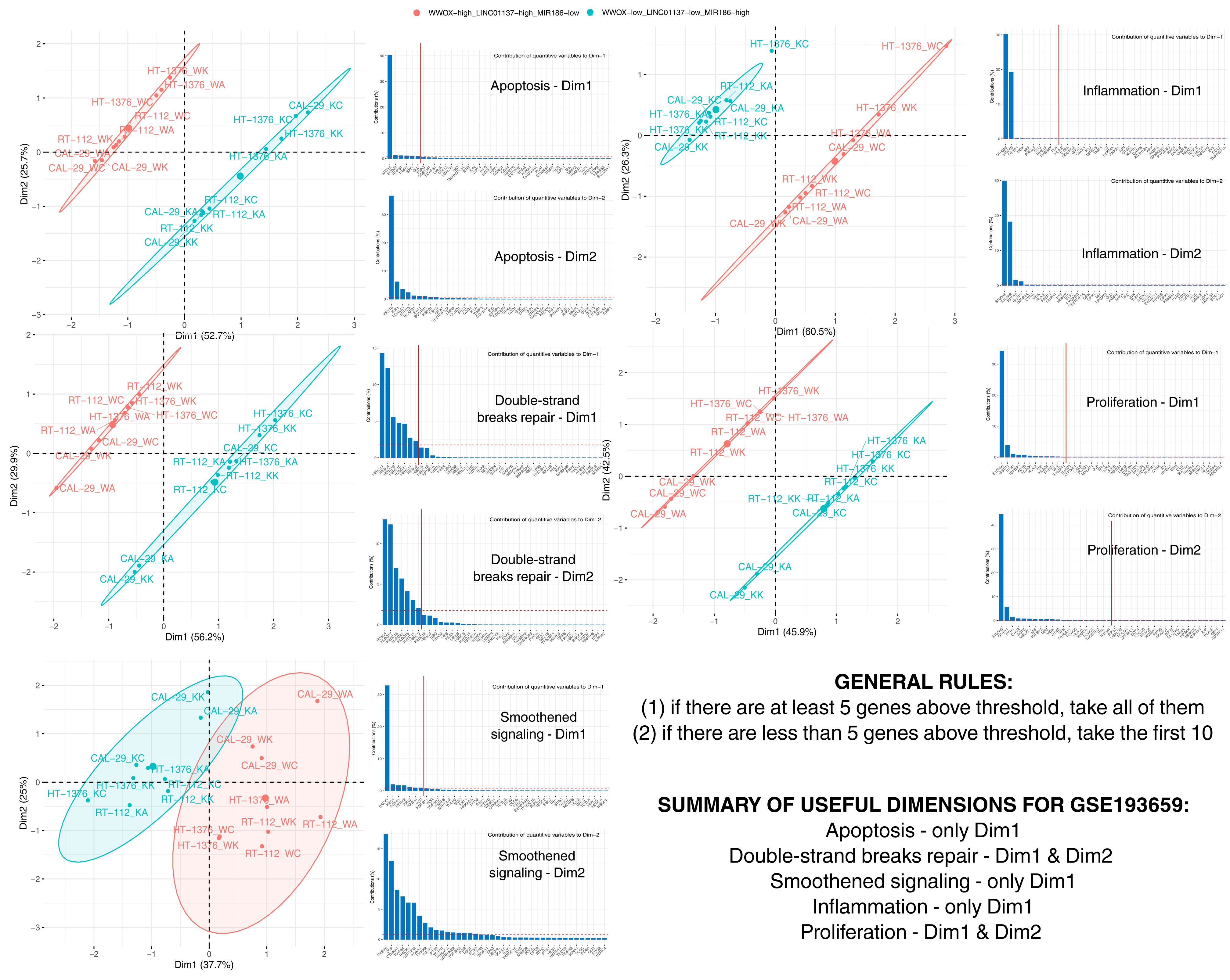


**Supplementary Figure 5.** Subsidiary MFA for GSE193659.


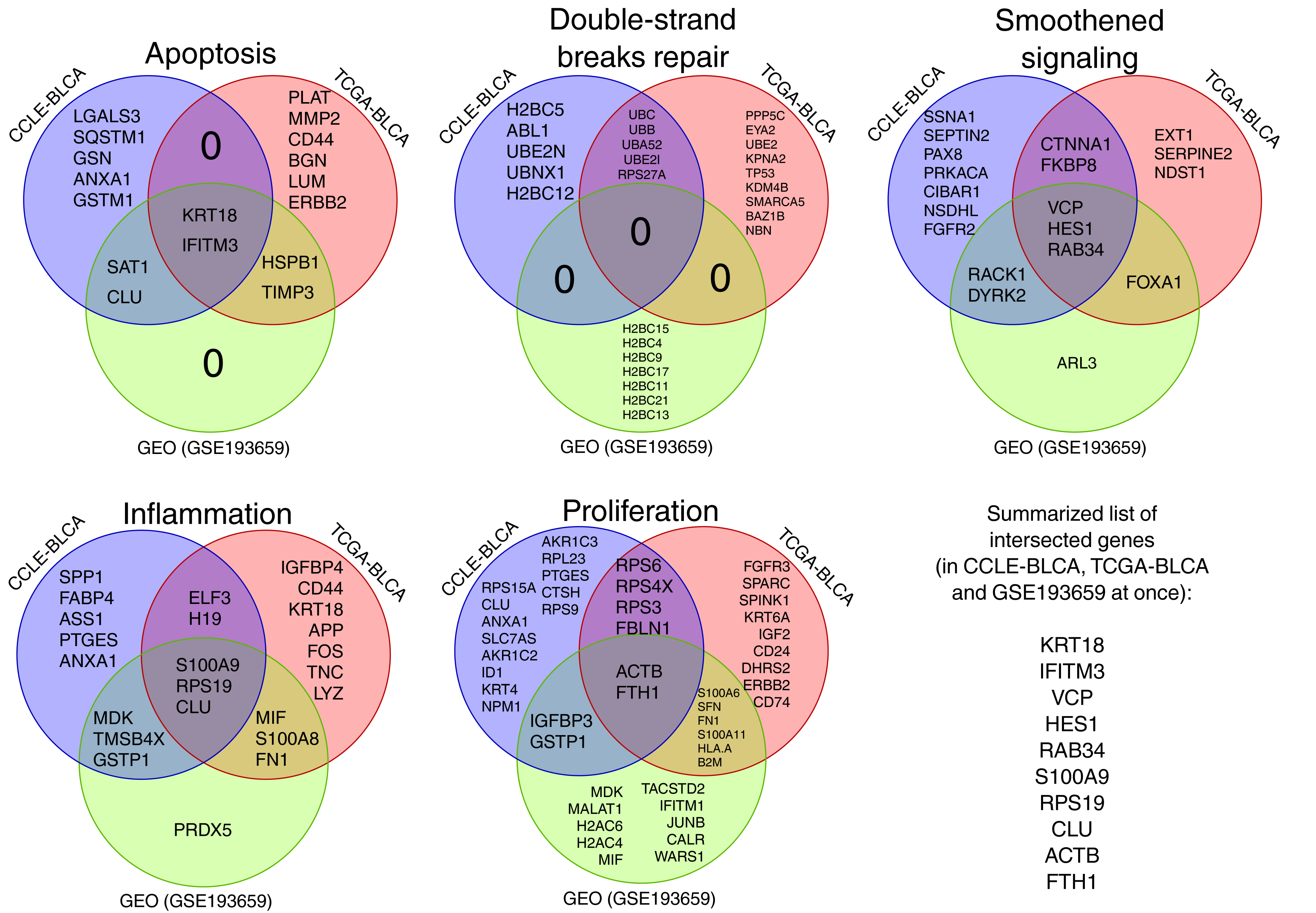


**Supplementary Figure 6.** Intersection analysis for subsidiary MFAs.


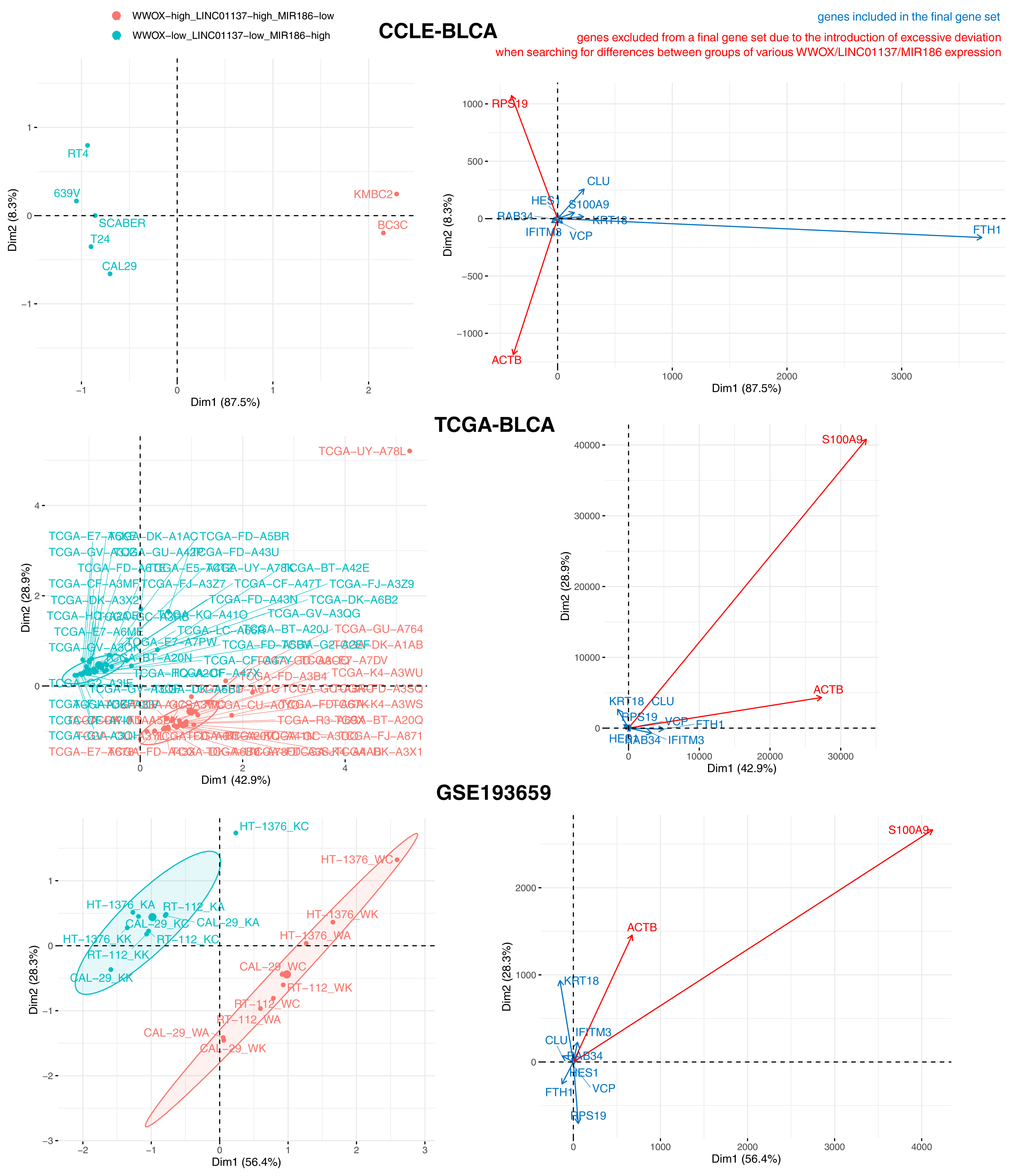


**Supplementary Figure 7.** Evaluation of intersected genes for their appropriateness in differentiating expression profiles.


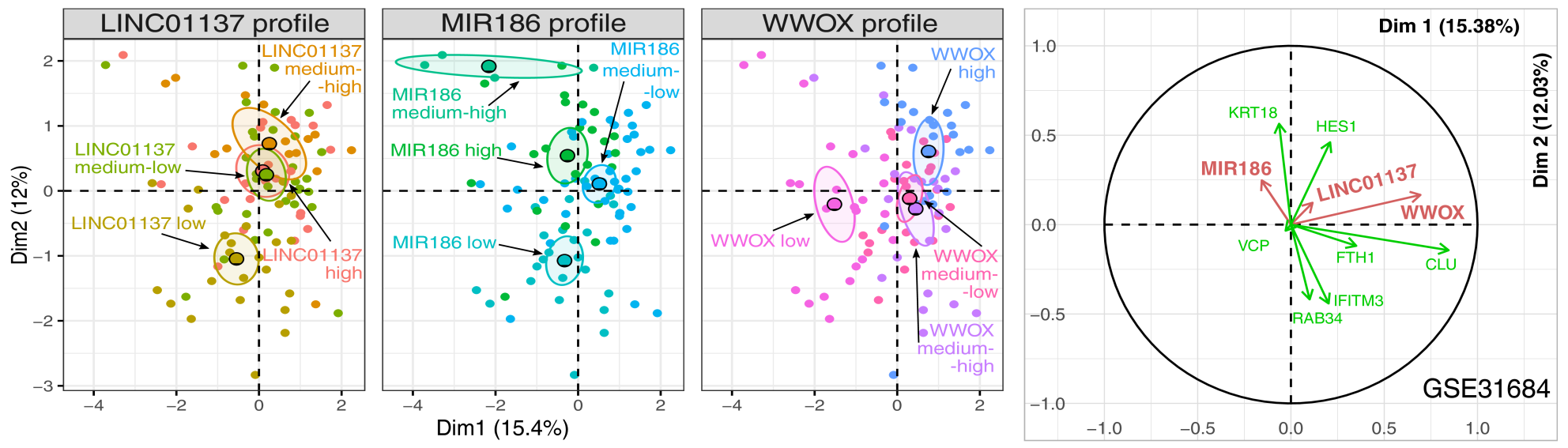


**Supplementary Figure 8.** Validation of definitive MFA using GSE31684.


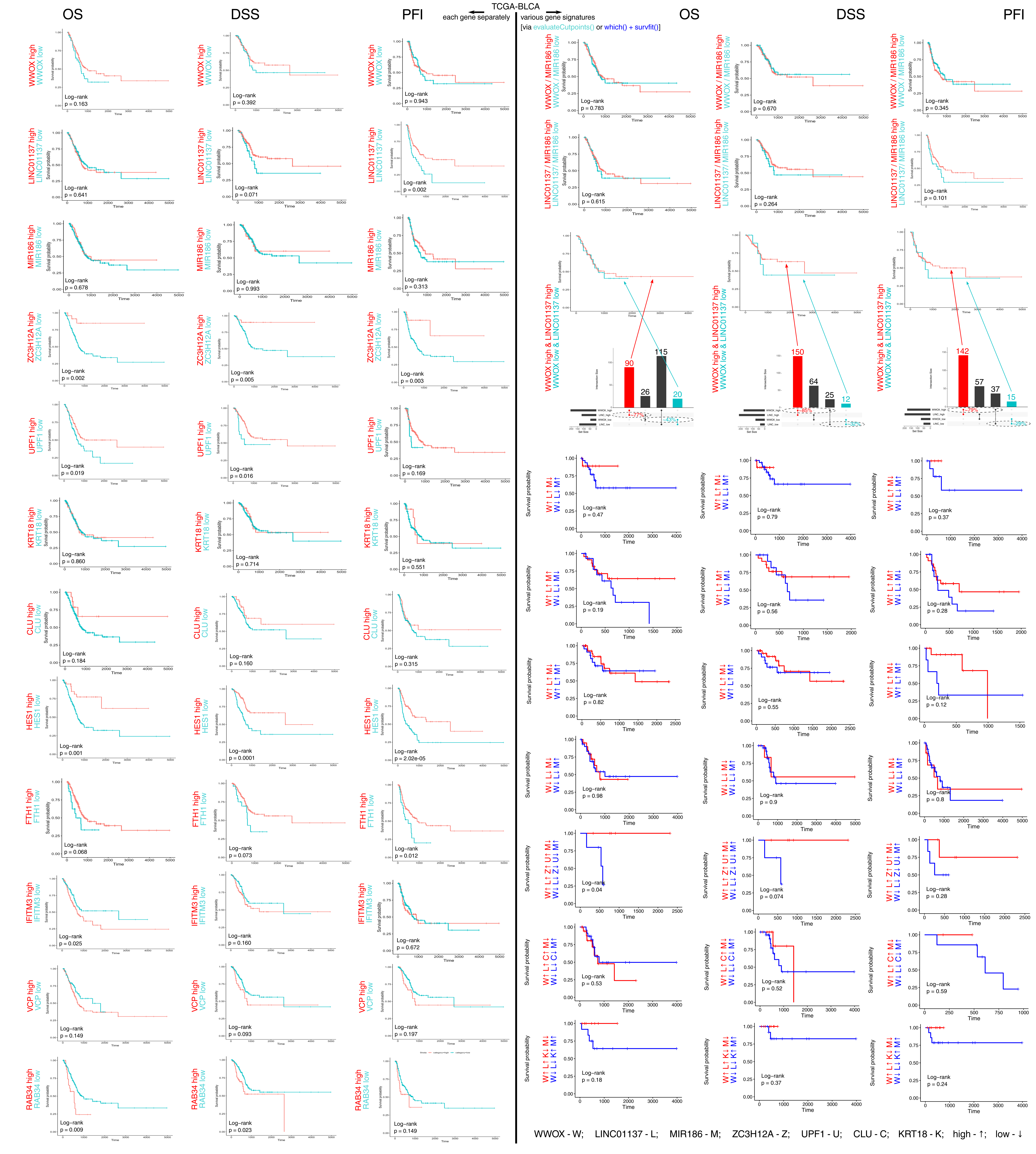


**Supplementary Figure 9.** Complete set of survival curves for patients from TCGA-BLCA.


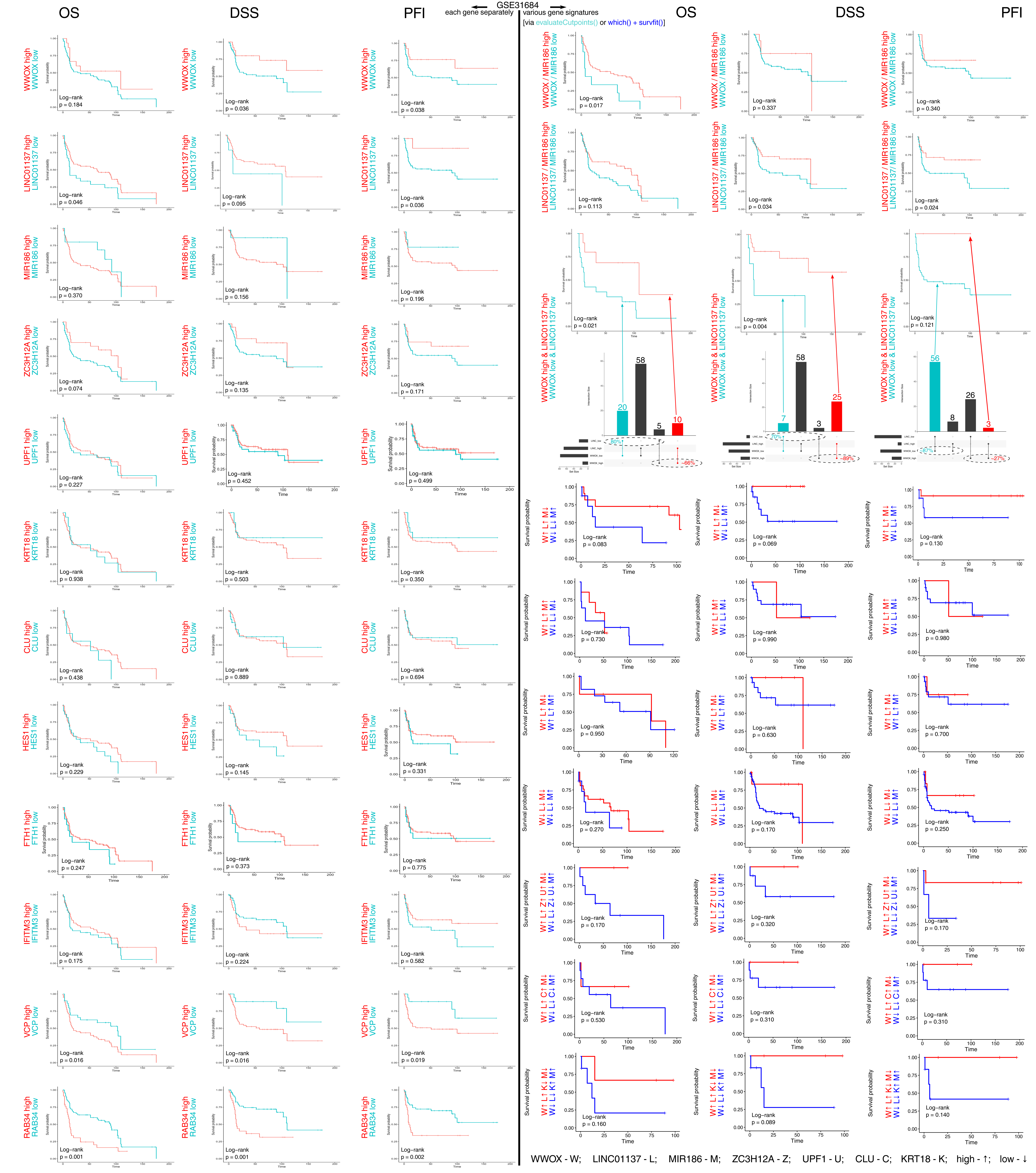


**Supplementary Figure 10.** Complete set of survival curves for patients from GSE31684.

## Supplementary Tables

**Supplementary Table 1.** Primers used in the study.

| **Gene** | **Forward primer sequence (5’ 🡪 3’)** | **Reverse primer sequence (5’ 🡪 3’)** |
| --- | --- | --- |
| Reference genes | | |
| *H3F3A* | ACGGATTACACCTTCCCACTTGCTAAAAGGTC | AGCCACAAAGGCAGATGGATCAGCCAA |
| *RPLP0* | AGGACTTTAAAAGATCTGCGCTTCCAGAG | ACCAGATAGGCCTCACTTGCCTCCTGC |
| *RPS17* | AAGCGCGTGTGCGAGGAGATCG | TCGCTTCATCAGATGCGTGACATAACCTG |
| *U6* | AAATTGGAACGATACAGAGAAGATTAGC | TATGGAACGCTTCACGAATTTGC |
| Investigated genes | | |
| *UPF1* | GCATGACTGCCGATGAATATGG | GCTGCTGTTGTTGTTGTTGC |
| *RBM22* | GTGAACATTGCTCTGCCACCG | ACATGTGTGGCCCAAAACCTGG |
| *ZC3H12A* | CGTAAGAAGCCACTCACTTTGG | GGTGGAAGAATCGGCACTTG |
| *MIR6732* | TAGGGGGTGGCAGGCTGG | GAACATGTCTGCGTATCTC |
| *LINC01137* | TCGGACCTAAGAACTTGACAGG | GAAACTTCGTCTCTGATTCACTCG |
| *MIR186* | AGAATTCTCCTTTTGGGC | GAACATGTCTGCGTATCTC |

**Supplementary Table 2.** Summary of gene ontology.

| **Functional annotation** | **No. of genes from query** | **Statistical significance** |
| --- | --- | --- |
| **GOTERM_BP_DIRECT (DAVID)** | | |
| gene silencing by miRNA | 111 | 1.80E-131 |
| miRNA mediated inhibition of translation | 29 | 2.20E-34 |
| negative regulation of cell migration involved in sprouting angiogenesis | 15 | 4.50E-17 |
| negative regulation of vascular endothelial growth factor production | 13 | 1.80E-16 |
| negative regulation of angiogenesis | 19 | 1.40E-15 |
| negative regulation of blood vessel endothelial cell proliferation involved in sprouting angiogenesis | 12 | 4.10E-15 |
| negative regulation of vascular endothelial cell proliferation | 10 | 1.60E-11 |
| negative regulation of cell migration | 16 | 2.10E-09 |
| positive regulation of vascular smooth muscle cell proliferation | 10 | 3.80E-08 |
| negative regulation of cytokine production involved in inflammatory response | 9 | 5.00E-08 |
| negative regulation of leukocyte adhesion to vascular endothelial cell | 6 | 8.60E-08 |
| mRNA cleavage involved in gene silencing by miRNA | 8 | 9.00E-08 |
| negative regulation of gene expression | 17 | 2.50E-07 |
| negative regulation of interleukin-8 production | 8 | 8.70E-07 |
| carbohydrate transport | 8 | 8.70E-07 |
| negative regulation of blood vessel endothelial cell migration | 8 | 9.80E-07 |
| negative regulation of vascular smooth muscle cell proliferation | 8 | 2.50E-06 |
| negative regulation of cell proliferation | 18 | 6.70E-06 |
| positive regulation of blood vessel endothelial cell proliferation involved in sprouting angiogenesis | 6 | 0.000021 |
| negative regulation of interleukin-6-mediated signaling pathway | 5 | 0.000022 |
| negative regulation of transporter activity | 6 | 0.00003 |
| negative regulation of inflammatory response | 10 | 0.00003 |
| positive regulation of connective tissue replacement | 5 | 0.00003 |
| negative regulation of vascular associated smooth muscle cell migration | 6 | 0.00005 |
| negative regulation of cell cycle G1/S phase transition | 5 | 0.00014 |
| negative regulation of SMAD protein import into nucleus | 5 | 0.00014 |
| negative regulation of G1/S transition of mitotic cell cycle | 7 | 0.00014 |
| positive regulation of cell migration involved in sprouting angiogenesis | 6 | 0.00016 |
| negative regulation of protein kinase B signaling | 7 | 0.00016 |
| positive regulation of cardiac muscle cell apoptotic process | 5 | 0.00039 |
| negative regulation of cholesterol efflux | 5 | 0.00046 |
| cellular response to glucose stimulus | 7 | 0.0012 |
| negative regulation of sprouting angiogenesis | 5 | 0.0012 |
| negative regulation of I-kappaB kinase/NF-kappaB signaling | 6 | 0.0012 |
| positive regulation of apoptotic process | 12 | 0.0012 |
| negative regulation of beta-amyloid formation | 5 | 0.0012 |
| negative regulation of drug transmembrane export | 4 | 0.002 |
| negative regulation of cell adhesion molecule production | 4 | 0.0031 |
| negative regulation of BMP signaling pathway | 6 | 0.0035 |
| negative regulation of interleukin-6 production | 6 | 0.0039 |
| negative regulation of endothelial cell proliferation | 5 | 0.0039 |
| positive regulation of vascular endothelial growth factor receptor signaling pathway | 4 | 0.0049 |
| negative regulation of fibroblast growth factor receptor signaling pathway | 4 | 0.0089 |
| positive regulation of cellular senescence | 4 | 0.0099 |
| negative regulation of BMP secretion | 3 | 0.015 |
| negative regulation of metalloendopeptidase activity | 3 | 0.015 |
| negative regulation of amyloid precursor protein catabolic process | 3 | 0.015 |
| positive regulation of vascular associated smooth muscle cell migration | 4 | 0.018 |
| negative regulation of vascular smooth muscle cell differentiation | 3 | 0.025 |
| negative regulation of insulin receptor signaling pathway | 4 | 0.047 |
| **GOTERM_CC_DIRECT (DAVID)** | | |
| RISC complex | 89 | 8.90E-112 |
| extracellular vesicle | 29 | 4.80E-30 |
| extracellular space | 58 | 1.10E-20 |
| **GOTERM_MF_DIRECT (DAVID)** | | |
| mRNA binding involved in posttranscriptional gene silencing | 73 | 1.40E-105 |
| mRNA 3'-UTR binding | 44 | 2.00E-52 |
| pyrimidine nucleotide-sugar transmembrane transporter activity | 8 | 2.00E-13 |
| **KEGG_PATHWAY (DAVID)** | | |
| MicroRNAs in cancer | 45 | 2.40E-53 |
| Chemical carcinogenesis - receptor activation | 10 | 2.30E-04 |
| **REACTOME_PATHWAY (DAVID)** |  |  |
| Negative regulation of activity of TFAP2 (AP-2) family transcription factors | 3 | 2.40E-02 |
| Activation of the TFAP2 (AP-2) family of transcription factors | 3 | 2.40E-02 |
| **WIKIPATHWAYS (DAVID)** | | |
| miRNAs involved in DNA damage response | 19 | 2.20E-20 |
| Cell differentiation - index | 12 | 2.50E-11 |
| Parkinson's disease pathway | 13 | 4.00E-11 |
| Interactions between immune cells and microRNAs in tumor microenvironment | 11 | 2.80E-10 |
| Cell differentiation - expanded index | 11 | 2.00E-09 |
| Metastatic brain tumor | 8 | 2.80E-08 |
| miRNA regulation of DNA damage response | 11 | 2.20E-07 |
| OSX and miRNAs in tooth development | 8 | 2.20E-07 |
| miRNA targets in ECM and membrane receptors | 8 | 1.10E-06 |
| miRNAs involvement in the immune response in sepsis | 9 | 1.10E-06 |
| let-7 inhibition of ES cell reprogramming | 6 | 1.30E-06 |
| Aspirin and miRNAs | 5 | 0.00014 |
| Hematopoietic stem cell differentiation | 6 | 0.002 |
| MicroRNAs in cardiomyocyte hypertrophy | 6 | 0.017 |
| MicroRNA network associated with chronic lymphocytic leukemia | 3 | 0.021 |
| **PANTHER Pathways (PANTHER)** | | |
| P53 pathway feedback loops 1 | 1 | 1.39E-02 |
| TCA cycle | 1 | 2.08E-02 |
| Heterotrimeric G-protein signaling pathway-Gi alpha and Gs alpha mediated pathway | 2 | 3.36E-02 |
| Endogenous cannabinoid signaling | 1 | 4.28E-02 |

**Supplementary Table 3.** Summary of survival analysis.

|  | **GEO (GSE31684)** | | | **TCGA-BLCA** | | |
| --- | --- | --- | --- | --- | --- | --- |
| **Gene or signature** | **OS** | **DSS** | **PFI** | **OS** | **DSS** | **PFI** |
| **WWOX** (abbr. „W”)  (high vs low) | 0.608 (0.29, 1.28) | 0.424* (0.19, 0.97) | 0.430* (0.18, 0.98) | 0.726 (0.46, 1.14) | 0.808 (0.49, 1.32) | 1.020 (0.66, 1.55) |
| **LINC01137** (abbr. „L”)  (high vs low) | 0.585* (0.34, 0.99) | 0.481 (0.20, 1.16) | 0.157* (0.02, 1.15) | 1.100 (0.75, 1.61) | 0.593 (0.34, 1.05) | 0.518** (0.34, 0.79) |
| **MIR186** (abbr. „M”)  (high vs low) | 1.470 (0.63, 3.41) | 2.700 (0.64, 11.2) | 2.480 (0.59, 10.3) | 0.908 (0.58, 1.43) | 0.997 (0.49, 2.01) | 0.820 (0.56, 1.21) |
| **ZC3H12A** (abbr. „Z”)  (high vs low) | 0.557 (0.29, 1.07) | 0.520 (0.22, 1.25) | 0.548 (0.23, 1.31) | 0.201** (0.06, 0.63) | 0.220** (0.07, 0.70) | 0.248** (0.09, 0.67) |
| **UPF1** (abbr. „U”)  (high vs low) | 0.726 (0.43, 1.22) | 1.490 (0.78, 2.84) | 1.600 (0.76, 3.40) | 0.630* (0.43, 0.93) | 0.445* (0.23, 0.87) | 0.585 (0.27, 1.27) |
| **KRT18** (abbr. „K”)  (high vs low) | 0.974 (0.51, 1.87) | 1.380 (0.54, 3.53) | 1.560 (0.61, 4.00) | 0.961 (0.62, 1.50) | 0.886 (0.46, 1.69) | 0.761 (0.31, 1.87) |
| **CLU** (abbr. „C”)  (high vs low) | 0.732 (0.33, 1.62) | 1.050 (0.49, 2.23) | 1.140 (0.58, 2.24) | 0.513 (0.19, 1.40) | 0.632 (0.33, 1.20) | 0.775 (0.47, 1.28) |
| **HES1**  (high vs low) | 0.708 (0.40, 1.25) | 0.585 (0.28, 1.21) | 0.700 (0.34, 1.44) | 0.307*** (0.15, 0.63) | 0.403*** (0.25, 0.65) | 0.438*** (0.29, 0.65) |
| **FTH1**  (high vs low) | 0.659 (0.32, 1.34) | 0.625 (0.22, 1.77) | 0.880 (0.37, 2.11) | 0.574 (0.32, 1.05) | 0.558 (0.29, 1.06) | 0.524* (0.32, 0.87) |
| **IFITM3**  (high vs low) | 0.712 (0.43, 1.17) | 1.500 (0.78, 2.92) | 0.582 (0.31, 1.10) | 1.560* (1.05, 2.30) | 1.400 (0.87, 2.25) | 1.090 (0.73, 1.64) |
| **VCP**  (high vs low) | 2.250* (1.14, 4.45) | 3.340* (1.18, 9.47) | 3.250* (1.15, 9.18) | 1.390 (0.89, 2.16) | 1.580 (0.92, 2.70) | 1.360 (0.85, 2.16) |
| **RAB34**  (high vs low) | 2.280*** (1.39, 3.76) | 2.830*** (1.48, 5.39) | 2.620** (1.38, 4.98) | 2.090** (1.18, 3.69) | 1.770* (1.07, 2.93) | 1.530 (0.85, 2.76) |
| **WWOX/MIR186**  (high ratio vs low ratio) | 0.433* (0.21, 0.88) | 0.605 (0.21, 1.71) | 0.607 (0.22, 1.71) | 0.946 (0.64, 1.40) | 1.110 (0.68, 1.81) | 1.200 (0.82, 1.77) |
| **LINC01137/MIR186**  (high ratio vs low ratio) | 0.863 (0.39, 1.11) | 0.475* (0.23, 0.96) | 0.453* (0.22, 0.92) | 0.882 (0.54, 1.44) | 0.728 (0.42, 1.27) | 0.683 (0.43, 1.08) |
| **WWOX & LINC01137**  (high&high vs low&low) | 0.292* (0.09, 0.88) | 0.190** (0.05, 0.66) | Log-rank p = 0.121 | 0.933 (0.46, 1.90) | 0.760 (0.27, 2.15) | 0.789 (0.34, 1.84) |
| **W↑ L↑ M↓** vs  **W↓ L↓ M↑** | 0.30  (0.07, 1.27) | Log-rank  p = 0.069 | 0.21  (0.02, 1.98) | 0.48  (0.06, 3.73) | 0.75  (0.09, 6.21) | Log-rank  p = 0.37 |
| **W↑ L↑ M↑** vs  **W↓ L↓ M↓** | 0.80  (0.23, 2.77) | 1.01  (0.12, 8.32) | 1.02  (0.12, 8.42) | 0.52  (0.19, 1.41) | 0.71  (0.23, 2.22) | 0.61  (0.25, 1.50) |
| **W↑ L↑ M↓** vs  **W↑ L↑ M↑** | 0.95  (0.22, 4.12) | 0.59  (0.07, 5.09) | 0.66  (0.08, 5.64) | 0.89  (0.33, 2.40) | 0.71  (0.23, 2.20) | 0.32  (0.07, 1.45) |
| **W↓ L↓ M↓** vs  **W↓ L↓ M↑** | 0.56  (0.19, 1.62) | 0.37  (0.09, 1.60) | 0.45  (0.11, 1.88) | 1.04  (0.36, 3.02) | 0.90  (0.19, 4.20) | 1.16  (0.38, 3.51) |
| **W↑ L↑ Z↑ U↑ M↓** vs  **W↓ L↓ Z↓ U↓ M↑** | Log-rank  p = 0.17 | Log-rank  p = 0.32 | 0.22  (0.02, 2.42) | Log-rank  p = 0.04 (*) | Log-rank  p = 0.074 | 0.30  (0.03, 2.95) |
| **W↑ L↑ C↑ M↓** vs  **W↓ L↓ C↓ M↑** | 0.52  (0.06, 4.55) | Log-rank  p = 0.31 | Log-rank  p = 0.31 | 1.37  (0.51, 3.69) | 0.61  (0.13, 2.89) | Log-rank  p = 0.59 |
| **W↑ L↑ K↓ M↓** vs  **W↓ L↓ K↑ M↑** | 0.24  (0.03, 2.20) | Log-rank  p = 0.089 | Log-rank  p = 0.14 | Log-rank  p = 0.18 | Log-rank  p = 0.37 | Log-rank  p = 0.24 |

High expression is favorable (statistical significance is met: p<0.05 [*]; p<0.01 [**]; p<0.001 [***])
High expression is favorable (visible trend without statistical significance)
High expression is unfavorable (statistical significance is met: p<0.05 [*]; p<0.01 [**]; p<0.001 [***])
High expression is unfavorable (visible trend without statistical significance)
For some comparisons, a dichotomous variable in one of the groups has no events.
Instead, log-rank p is provided.
